# Supplementary figures and images for: Assessment of transparency indicators across the biomedical literature: How open is open?
Source: PLoS Biol. 2021 Mar 1;19(3):e3001107. doi: 10.1371/journal.pbio.3001107 (PMC7951980; doi:10.1371/journal.pbio.3001107)

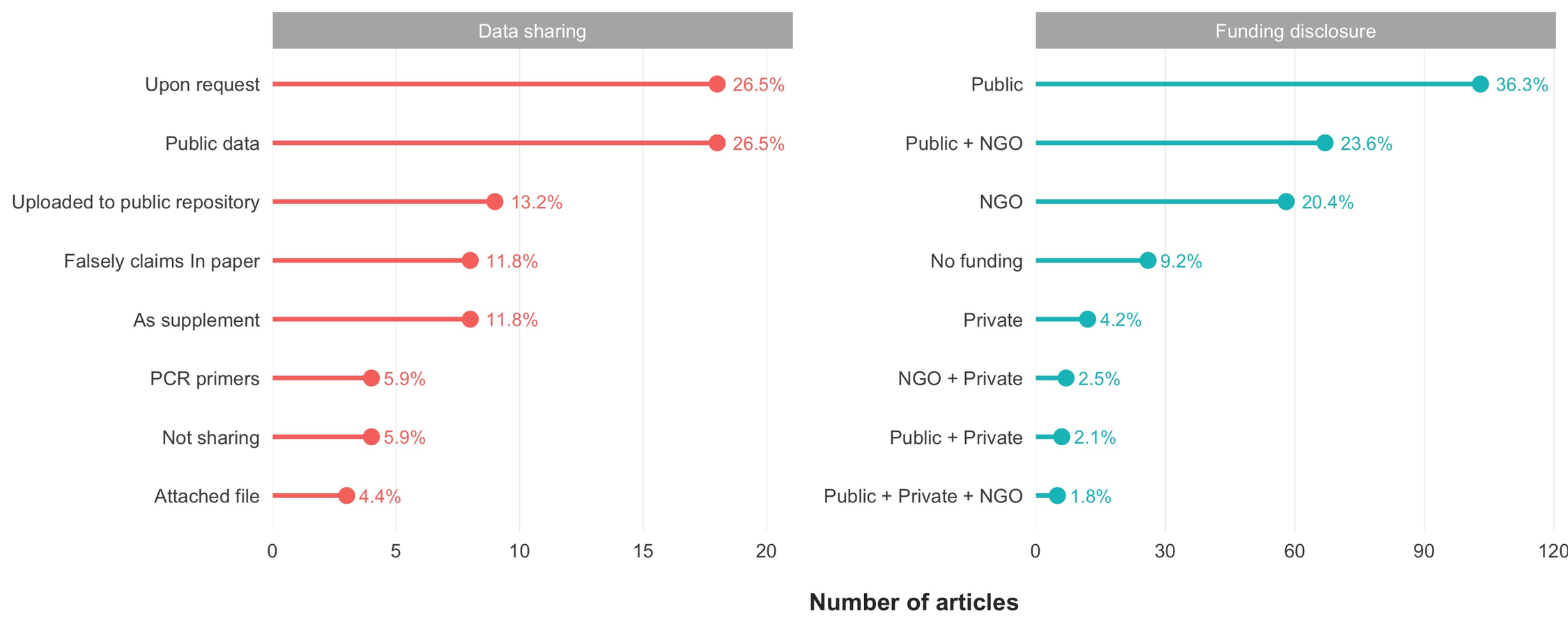

Supplement: S1 Fig — Of 68 research articles with Data sharing statements, most claimed availability upon request or made use of public data. Of those actively sharing new data, most made their data available on an online repository (e.g., GenBank); 8 articles stated that all of their data were available in the text or supplements, but we could not locate any such raw data—all 8 were published in PLOS ONE; 4 articles only shared PCR primers; 4 articles actively indicated that they are not currently sharing their data. Of 284 research articles with Funding disclosures, most reported public funds (e.g., NIH) or funds from NGOs (e.g., Gates Foundation). Very few indicated no or private funding. The data underlying this figure can be found on OSF at http://www.doi.org/10.17605/OSF.IO/E58WS. NGO, Non-Governmental Organization; NIH, National Institutes of Health; OSF, Open Science Framework. (TIF) [file pbio.3001107.s001.tif]

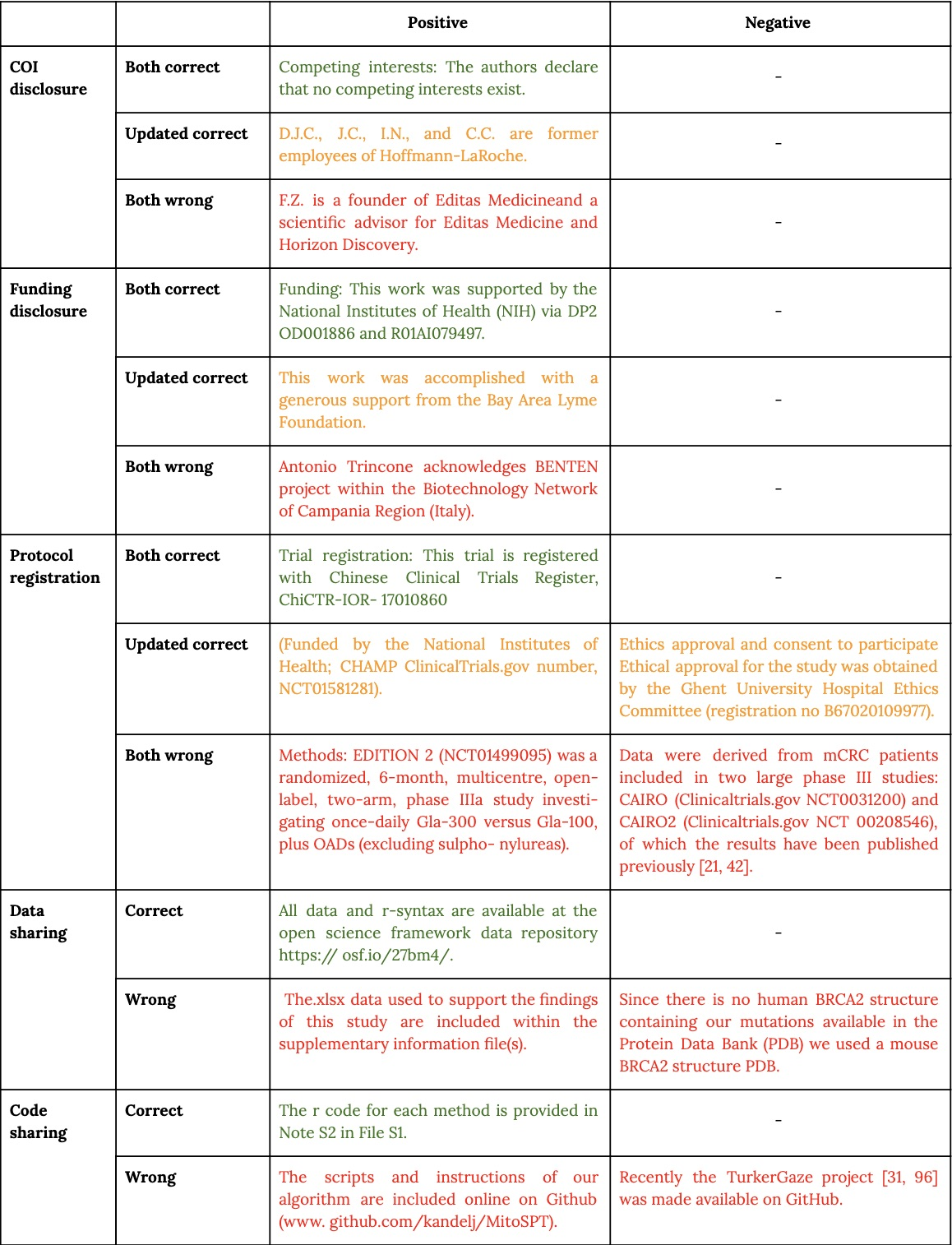

Supplement: S2 Fig — This figure illustrates examples of text predicted Positive (i.e., containing the indicator of interest) or Negative (i.e., not containing the indicator of interest) by the algorithm developed in the initial sample of 499 articles and the updated algorithm using data from the 6,017 articles. Both correct, this text was labeled correctly by both algorithms; Updated correct, this text was labeled correctly only by the updated algorithm; Both wrong, this text that was labeled incorrectly by both algorithms. Notice that the green statements for COI disclosures, Funding disclosures, and Protocol registration are very explicit about their content, the orange statements slightly less so, and the red statements even less explicit—this illustrates how the algorithms were updated to capture more of the less explicit statements (see S2 Text). Note that these statements were purposefully selected because they are small and clearly exemplify the points made—to access the complete evaluation of these algorithms and all sentences classified correctly or incorrectly, please see our data on OSF at http://www.doi.org/10.17605/OSF.IO/E58WS. COI, Conflict of interest; OSF, Open Science Framework. (TIF) [file pbio.3001107.s002.tif]

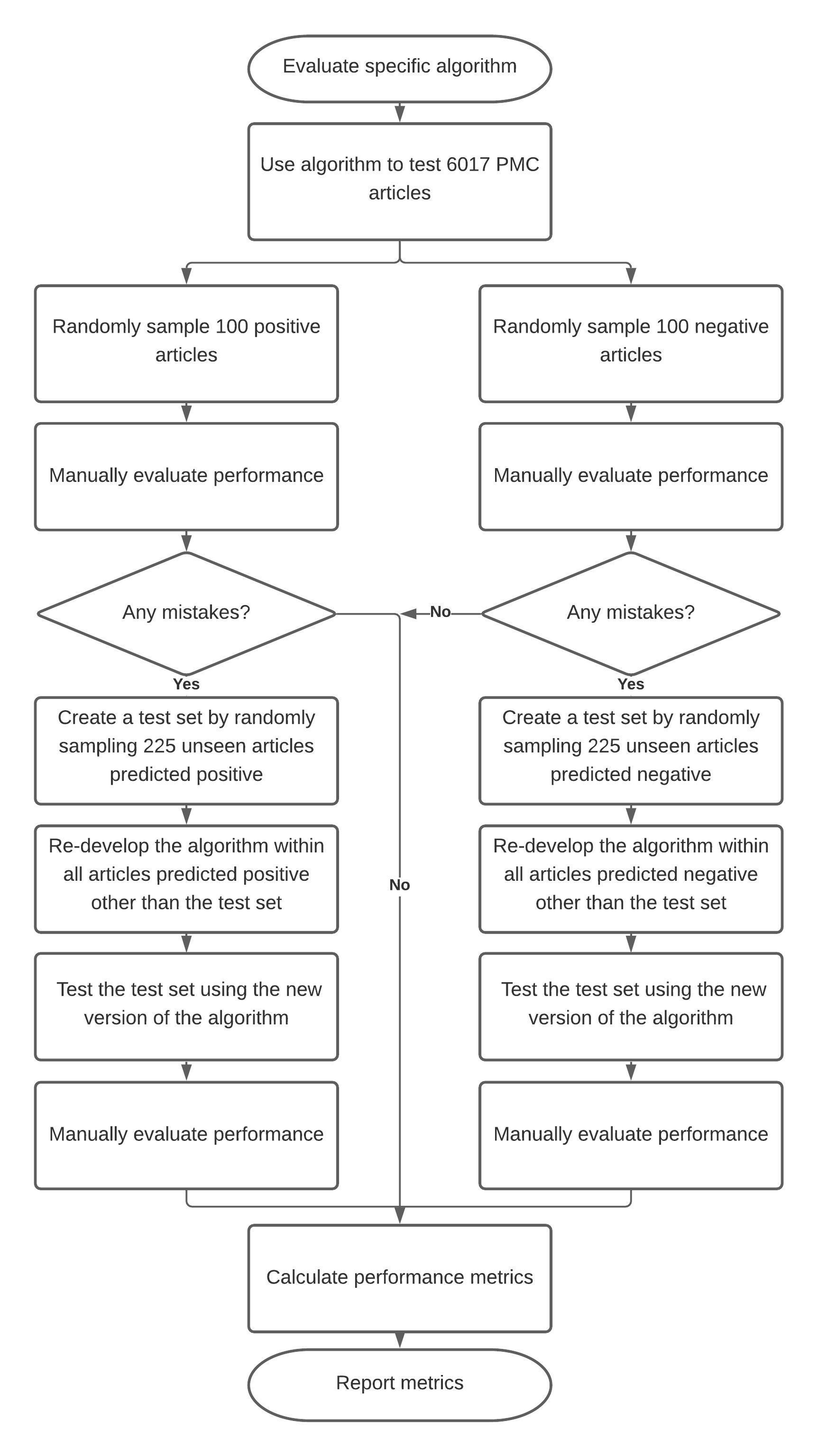

Supplement: S3 Fig — A flowchart illustrating the basic outline of our approach to validating our algorithms. (TIF) [file pbio.3001107.s003.tif]
