# Supplementary material for: Assessment of transparency indicators across the biomedical literature: How open is open?
Source: PLoS Biol. 2021 Mar 1;19(3):e3001107. doi: 10.1371/journal.pbio.3001107 (PMC7951980; doi:10.1371/journal.pbio.3001107)
Supplement: S1 Table — Novelty or Replication statement, language suggesting that the authors are claiming novelty and/or replication (Yes), or neither (No). COI, Conflict of interest; Full-text, full-text article; PubMed, the PubMed record of an article. (DOCX) [file pbio.3001107.s004.docx]

**S1 Table. Indicator identification using the full-text vs the PubMed record of an article from PubMed published between 2015-2018.**

| **Indicator** |  | **Full-text** | **PubMed** |
| --- | --- | --- | --- |
|  |  | **N = 499 articles** | |
| **COI disclosure** | **Yes** | 341 (68%) | 36 (7%) |
|  | **No** | 158 (32%) | 463 (93%) |
| **Funding disclosure** | **Yes** | 352 (71%) | 177 (36%) |
|  | **No** | 147 (29%) | 322 (64%) |
|  | | | |
|  |  | **n = 349 research articles** | |
| **Data sharing** | **Yes** | 68 (20%) | 3 (1%) |
|  | **No** | 281 (80%) | 346 (99%) |
| **Code sharing** | **Yes** | 5 (1%) | 0 (0%) |
|  | **No** | 344 (99%) | 349 (100%) |
| **Protocol registration** | **Yes** | 22 (6%) | 12 (3%) |
|  | **No** | 327 (94%) | 337 (97%) |
| **Novelty or Replication statement** | **Yes** | 193 (55%) | 110 (32%) |
|  | **No** | 156 (45%) | 239 (69%) |
