# Supplementary material for: Assessment of transparency indicators across the biomedical literature: How open is open?
Source: PLoS Biol. 2021 Mar 1;19(3):e3001107. doi: 10.1371/journal.pbio.3001107 (PMC7951980; doi:10.1371/journal.pbio.3001107)
Supplement: S2 Table — COI, Conflict of interest; N, number of all articles; n, number of research articles; NGO, Non-Governmental Organization. (DOCX) [file pbio.3001107.s005.docx]

**S2 Table. Indicators of transparency across three different random PubMed samples studying articles from 2000-2014, 2015-2017 and 2015-2018 (current publication).**

| **Indicator** |  |  | **2000-2014** | **2015-2017** | **2015-2018** |
| --- | --- | --- | --- | --- | --- |
| **All articles** |  |  | **N = 442** | **N = 148** | **N = 499** |
| **COI disclosure** | **Yes** | **Reported no presence of conflicts** | 110 (24.9%) | 87 (58.8%) | 301 (60.3%) |
|  |  | **Reported conflicts** | 26 (5.9%) | 10 (6.8%) | 40 (8.0%) |
|  | **No** |  | 306 (69.2%) | 51 (34.5%) | 158 (31.7%) |
| **Funding disclosure** | **Yes** | **Public** | 87 (19.7%) | 55 (37.2%) | 122 (24.4%) |
|  |  | **Public + Private** | 9 (2.0%) | 2 (1.4%) | 6 (1.2%) |
|  |  | **Public + Private + NGO** | 4 (0.9%) | 3 (2.0%) | 6 (1.2%) |
|  |  | **Public + NGO** | 54 (12.2%) | 18 (12.2%) | 79 (15.8%) |
|  |  | **Private** | 19 (4.3%) | 2 (1.4%) | 12 (2.4%) |
|  |  | **No funding** | 12 (2.7%) | 10 (6.8%) | 46 (9.2%) |
|  |  | **NGO** | 29 (6.6%) | 12 (8.1%) | 74 (14.8%) |
|  |  | **NGO + Private** | 1 (0.2%) | 1 (0.7%) | 7 (1.4%) |
|  | **No** |  | 227 (51.4%) | 45 (30.4%) | 147 (29.5%) |
|  | | | | | |
| **Research articles** |  |  | **n = 260** | **n = 95** | **n = 349** |
| **Code sharing** |  | **Yes** | 0 (0.0%) | 0 (0.0%) | 5 (1.4%) |
|  |  | **No** | 260 (100.0%) | 95 (100.0%) | 344 (98.6%) |
| **Data sharing** |  | **Yes** | 5 (1.9%) | 19 (20.0%) | 68 (19.5%) |
|  |  | **No** | 255 (98.1%) | 76 (80.0%) | 281 (80.5%) |
| **Registration** |  | **Yes** | 6 (2.3%) | 31 (32.6%) | 22 (6.3%) |
|  |  | **No** | 254 (97.7%) | 64 (67.4%) | 327 (93.7%) |
| **Novelty** |  | **Yes** | 139 (53.5%) | 64 (67.4%) | 175 (50.1%) |
|  |  | **No** | 121 (46.5%) | 31 (32.6%) | 174 (49.9%) |
| **Replication** |  | **Yes** | 10 (3.8%) | 15 (15.8%) | 33 (9.5%) |
|  |  | **No** | 250 (96.2%) | 80 (84.2%) | 316 (90.5%) |
