# Supplementary material for: Assessment of transparency indicators across the biomedical literature: How open is open?
Source: PLoS Biol. 2021 Mar 1;19(3):e3001107. doi: 10.1371/journal.pbio.3001107 (PMC7951980; doi:10.1371/journal.pbio.3001107)
Supplement: S3 Table — p-Values were calculated using the Fisher exact test. Non-PMC, articles available on PubMed, but not PMC; PMC, articles available on PubMed and PMC; N, all articles; n, research articles. (DOCX) [file pbio.3001107.s006.docx]

**S3 Table. Indicator prevalence by presence or absence from PubMed Central.**

| **Indicator** |  | **non-PMC** | **PMC** | **P-value** |
| --- | --- | --- | --- | --- |
|  |  | **N = 197** | **N = 302** |  |
| **COI disclosure** | No | 37 (18.8%) | 121 (40.1%) | 4.4E-07 |
|  | Yes | 160 (81.2%) | 181 (59.9%) |  |
| **Funding disclosure** | No | 46 (23.4%) | 101 (33.4%) | 0.016 |
|  | Yes | 151 (76.6%) | 201 (66.6%) |  |
|  | | | | |
|  |  | **n = 145** | **n = 204** |  |
| **Data sharing** | No | 91 (62.8%) | 190 (93.1%) | 2.0E-12 |
|  | Yes | 54 (37.2%) | 14 (6.9%) |  |
| **Code sharing** | No | 141 (97.2%) | 203 (99.5%) | 0.16 |
|  | Yes | 4 (2.8%) | 1 (0.5%) |  |
| **Protocol registration** | No | 135 (93.1%) | 192 (94.1%) | 0.82 |
|  | Yes | 10 (6.9%) | 12 (5.9%) |  |
| **Novelty** | No | 71 (49.0%) | 103 (50.5%) | 0.83 |
|  | Yes | 74 (51.0%) | 101 (49.5%) |  |
| **Replication** | No | 134 (92.4%) | 182 (89.2%) | 0.36 |
|  | Yes | 11 (7.6%) | 22 (10.8%) |  |
