# Supplementary material for: Assessment of transparency indicators across the biomedical literature: How open is open?
Source: PLoS Biol. 2021 Mar 1;19(3):e3001107. doi: 10.1371/journal.pbio.3001107 (PMC7951980; doi:10.1371/journal.pbio.3001107)
Supplement: S6 Table — PMCOA, PubMed Central Open Access. (DOCX) [file pbio.3001107.s009.docx]

**S6 Table. The 10 most common combinations of indicator co-occurrence in all 2,751,420 open access PubMed Central (PMCOA) publications.**

| **Data** | **Code** | **COI** | **Funding** | **Protocol** | **Number** | **%** |
| --- | --- | --- | --- | --- | --- | --- |
| No | No | Yes | Yes | No | 1,273,382 | 46.3% |
| No | No | No | No | No | 535,428 | 19.5% |
| No | No | Yes | No | No | 339,233 | 12.3% |
| No | No | No | Yes | No | 278,282 | 10.1% |
| Yes | No | Yes | Yes | No | 172,393 | 6.3% |
| No | No | Yes | Yes | Yes | 58,456 | 2.1% |
| Yes | No | No | Yes | No | 38,515 | 1.4% |
| Yes | Yes | Yes | Yes | No | 16,020 | 0.6% |
| No | Yes | Yes | Yes | No | 11,426 | 0.4% |
| No | No | Yes | No | Yes | 5,722 | 0.2% |
