# Supplementary material for: Assessment of transparency indicators across the biomedical literature: How open is open?
Source: PLoS Biol. 2021 Mar 1;19(3):e3001107. doi: 10.1371/journal.pbio.3001107 (PMC7951980; doi:10.1371/journal.pbio.3001107)
Supplement: S7 Table — The p-value was calculated using the Kruskal–Wallis nonparametric test (tests whether samples originate from the same distribution)—for our data, this is equivalent to the Mann–Whitney U test. p-Values <10–16 have been replaced by <10–16. All values are per article. Citation count was taken from OCC and Journal Impact Factor of 2018 from WOS. OCC, Open Citation Collection; WOS, Web of Science. (DOCX) [file pbio.3001107.s010.docx]

**S7 Table. Meta-data across indicators from 2,498,496 articles published from 2000 onwards.**

| **Indicator** | **Variable** | **Absent** | **Present** | **P-value** |
| --- | --- | --- | --- | --- |
|  |  | **Median (IQR)** | **Median (IQR)** |  |
| **Data sharing** | **Citation count** | 6 (2-14) | 7 (3-17) | < 10^-16^ |
|  | **Journal Impact Factor** | 3 (2-4) | 3 (3-5) | < 10^-16^ |
|  | **Number of affiliations** | 3 (1-4) | 4 (2-5) | < 10^-16^ |
|  | **Number of authors** | 5 (3-8) | 6 (4-9) | < 10^-16^ |
|  | **Number of figures** | 3 (1-5) | 5 (3-7) | < 10^-16^ |
|  | **Number of references** | 34 (19-50) | 48 (34-65) | < 10^-16^ |
|  | **Number of tables** | 1 (0-3) | 2 (0-3) | < 10^-16^ |
|  | **Year** | 2016 (2013-2018) | 2017 (2015-2018) | < 10^-16^ |
| **Code sharing** | **Citation count** | 6 (2-15) | 6 (2-15) | 0.20 |
|  | **Journal Impact Factor** | 3 (2-4) | 4 (3-7) | < 10^-16^ |
|  | **Number of affiliations** | 3 (2-4) | 3 (2-5) | < 10^-16^ |
|  | **Number of authors** | 5 (3-8) | 5 (3-8) | 0.97 |
|  | **Number of figures** | 3 (1-5) | 5 (3-7) | < 10^-16^ |
|  | **Number of references** | 35 (20-52) | 46 (31-64) | < 10^-16^ |
|  | **Number of tables** | 1 (0-3) | 1 (0-3) | 3.5 x 10^-11^ |
|  | **Year** | 2016 (2013-2018) | 2018 (2016-2019) | < 10^-16^ |
| **COI disclosure** | **Citation count** | 7 (3-18) | 6 (2-14) | < 10^-16^ |
|  | **Journal Impact Factor** | 3 (2-5) | 3 (2-4) | < 10^-16^ |
|  | **Number of affiliations** | 2 (1-3) | 3 (2-4) | < 10^-16^ |
|  | **Number of authors** | 4 (3-7) | 5 (4-8) | < 10^-16^ |
|  | **Number of figures** | 2 (0-5) | 3 (1-6) | < 10^-16^ |
|  | **Number of references** | 22 (5-42) | 38 (25-54) | < 10^-16^ |
|  | **Number of tables** | 0 (0-2) | 1 (0-3) | < 10^-16^ |
|  | **Year** | 2014 (2010-2016) | 2016 (2014-2018) | < 10^-16^ |
| **Funding disclosure** | **Citation count** | 4 (2-11) | 6 (2-16) | < 10^-16^ |
|  | **Journal Impact Factor** | 3 (2-4) | 3 (3-4) | < 10^-16^ |
|  | **Number of affiliations** | 2 (1-3) | 3 (2-5) | < 10^-16^ |
|  | **Number of authors** | 4 (2-6) | 6 (4-8) | < 10^-16^ |
|  | **Number of figures** | 1 (0-3) | 4 (2-6) | < 10^-16^ |
|  | **Number of references** | 18 (5-34) | 40 (27-56) | < 10^-16^ |
|  | **Number of tables** | 0 (0-2) | 1 (0-3) | < 10^-16^ |
|  | **Year** | 2015 (2012-2017) | 2016 (2013-2018) | < 10^-16^ |
| **Protocol registration** | **Citation count** | 6 (2-15) | 6 (2-13) | 2.3 x 10^-9^ |
|  | **Journal Impact Factor** | 3 (2-4) | 3 (2-4) | < 10^-16^ |
|  | **Number of affiliations** | 3 (2-4) | 4 (3-7) | < 10^-16^ |
|  | **Number of authors** | 5 (3-8) | 7 (5-11) | < 10^-16^ |
|  | **Number of figures** | 3 (1-6) | 2 (1-3) | < 10^-16^ |
|  | **Number of references** | 35 (20-52) | 36 (27-49) | < 10^-16^ |
|  | **Number of tables** | 1 (0-3) | 3 (1-4) | < 10^-16^ |
|  | **Year** | 2016 (2013-2018) | 2017 (2015-2018) | < 10^-16^ |
