# Supplementary material for: Assessment of transparency indicators across the biomedical literature: How open is open?
Source: PLoS Biol. 2021 Mar 1;19(3):e3001107. doi: 10.1371/journal.pbio.3001107 (PMC7951980; doi:10.1371/journal.pbio.3001107)
Supplement: S8 Table — This table was created like so: First, we only kept clusters with at least 250 articles between 2015 and 2019. Then, we randomly sampled 3 clusters within each field. We then identified the name of each cluster, the most prevalent journal within that cluster, and the title of a representative review for each cluster. Each cluster, journal, and name within each field has been separated by a semi-colon. Two fields were excluded (EARTH and HUMANITIES) due to the 250 papers threshold—these fields have very little presence in PubMed. (DOCX) [file pbio.3001107.s011.docx]

**S8 Table. Representative clusters, journals and reviews for each field of science.**

| **Field** | **Clusters** | **Journals** | **Reviews** |
| --- | --- | --- | --- |
| **BIOLOGY** | snake venom composition; legged locomotion; silicon and plant growth | Toxicon; J Exp Biol; Front Plant Sci | Guiding recombinant antivenom development by omics technologies; What factors determine the preferred gait transition speed in humans? A review of the triggering mechanisms; Role of silicon in plant stress tolerance: opportunities to achieve a sustainable cropping system |
| **BRAIN** | intracranial aneurysms, MRA; computed tomography perfusion; transient ischemic attack | AJNR Am J Neuroradiol; Stroke; Stroke | MRA versus DSA for the follow-up imaging of intracranial aneurysms treated using endovascular techniques: a meta-analysis; Computed Tomography, Computed Tomography Angiography, and Perfusion Computed Tomography Evaluation of Acute Ischemic Stroke; Clinical Risk Score for Predicting Recurrence Following a Cerebral Ischemic Event |
| **CHEMISTRY** | microgel particles; coffee beans, bioactive compounds; H2O2 biosensing | Soft Matter; Food Chem; Biosens Bioelectron | Stimuli-Responsive Microgels and Microgel-Based Systems: Advances in the Exploitation of Microgel Colloidal Properties and Their Interfacial Activity; Furan in roasted, ground and brewed coffee; Quantitative analysis of hydrogen peroxide with special emphasis on biosensors |
| **COMP SCI** | image segmentation; adaptive dynamic programming; spatial frequency domain imaging | IEEE Trans Image Process; IEEE Trans Neural Netw Learn Syst; J Biomed Opt | A Survey of Graph Cuts/Graph Search Based Medical Image Segmentation; Distributed Estimation Techniques for Cyber-Physical Systems: A Systematic Review; Advances in the simulation of light-tissue interactions in biomedical engineering |
| **ENGNG** | lead bioaccessibility; droplet microfluidics; polycyclic aromatic hydrocarbons | Sci Total Environ; Lab Chip; Sci Total Environ | Oral Bioavailability of As, Pb, and Cd in Contaminated Soils, Dust, and Foods based on Animal Bioassays: A Review; Continuous magnetic droplets and microfluidics: generation, manipulation, synthesis and detection; Spatial distribution of polycyclic aromatic hydrocarbon contamination in urban soil of China |
| **HEALTH** | self-rated health; total hip arthroplasty; active school transportation | PLoS One; J Arthroplasty; J Phys Act Health | The effect of self-reported health on latent herpesvirus reactivation and inflammation in an ethnically diverse sample; A systematic review and meta-analysis of the direct anterior approach for hemiarthroplasty for femoral neck fracture; Effectiveness of active school transport interventions: a systematic review and update |
| **INF DIS** | Burkholderia mallei; bluetongue virus; ESBL-producing E. coli | PLoS Negl Trop Dis; Vet Ital; Front Microbiol | Melioidosis; Prospects of Next-Generation Vaccines for Bluetongue; Reviewing Interventions against Enterobacteriaceae in Broiler Processing: Using Old Techniques for Meeting the New Challenges of ESBL E |
| **MEDICINE** | colon mucus layer; avian influenza A; malignant hyperthermia | Sci Rep; J Infect; Anesthesiology | Fight them or feed them: how the intestinal mucus layer manages the gut microbiota; Did the Highly Pathogenic Avian Influenza A(H7N9) Viruses Emerged in China Raise Increased Threat to Public Health?; Malignant Hyperthermia in the Post-Genomics Era: New Perspectives on an Old Concept |
| **PHYS/MATH** | Gale crater; cavitation bubbles; sonocatalytic degradation | Astrobiology; Ultrason Sonochem; Ultrason Sonochem | Catalytic/Protective Properties of Martian Minerals and Implications for Possible Origin of Life on Mars; Using power ultrasound to accelerate food freezing processes: Effects on freezing efficiency and food microstructure; Hybrid Advanced Oxidation Processes Involving Ultrasound: An Overview |
| **SOC SCI** | prosocial behavior; peer victimization; causal mediation analysis | J Exp Child Psychol; J Interpers Violence; Epidemiology | The multidimensional nature of early prosocial behavior: a motivational perspective; Annual Research Review: The persistent and pervasive impact of being bullied in childhood and adolescence: implications for policy and practice; Can a Mediator Moderate? Considering the Role of Time and Change in the Mediator-Moderator Distinction |
