# Supplementary material for: Assessment of transparency indicators across the biomedical literature: How open is open?
Source: PLoS Biol. 2021 Mar 1;19(3):e3001107. doi: 10.1371/journal.pbio.3001107 (PMC7951980; doi:10.1371/journal.pbio.3001107)
Supplement: S9 Table — These phrases were chosen like so: First, a random sample of 10 articles was identified for each field-indicator pair (seed 1515). Then, the sample was ordered in terms of descending citation counts and was inspected from top to bottom for succinct representative phrases. The text was truncated to fit in 3 lines and any truncated text is denoted as “[…]”. The extracted text for “Code sharing” in Humanities is a false positive—2 (0.5%) texts from the Humanities were labeled as sharing code, both of which were false positives; nevertheless, both texts were highly relevant to code. For the code and data used to construct this table, see Data and Code Sharing statements. (DOCX) [file pbio.3001107.s012.docx]

**S9 Table. Representative text for each indicator of transparency across fields of science.**

| **Field** | **Data sharing** | **Code sharing** | **COI disclosure** | **Funding disclosure** | **Protocol registration** |
| --- | --- | --- | --- | --- | --- |
| **BIOLOGY** | the sequencing data of the five degradome libraries are available under ncbi-geo series accession [...] | software used in this manuscript is freely available at https://github com/smith-chem-wisc/gptmd [...] | Competing interests The authors declare that they have no competing interests. | This paper was supported by CNPq, CNPq/INCT-Doen‚àö√üas Tropicais, FAPEMIG, CAPES/PROCAD, and CAPES. | [...] a protocol was registered with the Prospero database (registration number CRD42013005307). |
| **BRAIN** | this data is available for download at the dbgap database (phs000092 v1 p1). | matlab scripts used in this analysis are available at: https://github com/vsevans/cest . | Competing Interests: The authors have declared that no competing interests exist. | This research received no specific grant from any funding agency in the public, commercial or not-for-profit sectors. | Trial registration This trial was registered with ClinicalTrials.gov, number NCT01716481. |
| **CHEMISTRY** | the data set is deposited in the gene expression omnibus (geo) database [ ] under accession no gse6997. | [...] this manuscript was produced with data and code archived at doi:10 5281/zenodo 3403173. [...] | The authors declare no competing financial interests. | Funding. This study was supported in part by the National Natural Science Foundation of China (31470407) [...] | Trial Registration ClinicalTrials.gov NCT01095848 |
| **COMP SCI** | [...] the datasets supporting the conclusions of the article are available in the dataverse [...] | bash and python scripts used to create the nfbs repository [...] are available on github at [...] | The authors declare that the research was conducted in the absence of any [...] conflict of interest. | The authors are grateful to [...] for supporting this research financially under Grants DIP-2012-03 [...] | The following systematic review was registered in PROSPERO with RN: CRD42019120058. |
| **EARTH** | our database ( supplementary data 1 ) consists of ~11500 5-min samples covering ~960 h dwell time [...] | the vpic code is a general-purpose pic simulation and available online ( https://github com/lanl/vpic ). | Competing interests The authors declare no competing financial interests. | Work at ICL was funded by STFC (UK) grant ST/N000692/1. [...] | - |
| **ENGNG** | s1 file (xlsx data availability section and the s4 file 10 1371/journal pone 0221363 | our code is available at: https://githubcom/raharjaliu/microtrench-chemotherapeutic-vision | Competing interests: The authors declare that they have no competing interests. | [...] thank the Alsace Region and the French-German Research Institute of Saint-Louis for funding this work. | Trial Registration Current Controlled Trials ISRCTN91381117 |
| **HEALTH** | raw data is attached to this article. | python code and associated search keywords are publicly available on a repository [...] | Conflicts of Interest: None declared. | This project is supported by General Research Fund of the Research Grants Council of Hong Kong (HKU 769408 M). | Systematic review registration PROSPERO 2015: CRD42015017327 |
| **INF DIS** | sequence data [...] have been deposited in genbank with the accession codes mf942137-mf942331. | code for these simulations is available at https://github com/braindynamicsusyd/spikenet | Competing interests The authors declare that they have no competing interests. | Funding. This work was supported by National Institutes of Health Grant CA 19014 (to NR-T). | Trial Registration ClinicalTrials.gov NCT00295581 |
| **HUMANITIES** | supplemental material available at figshare: https://doi org/10 25386/genetics6304502 . | the source code was highly valuable-arguably more so than the software itself-because [...] | The authors declare they have no competing financial interests. | Funding: The author received no specific funding for this article. | - |
| **MEDICINE** | all data are available from the geo accession numbers gse39040 gse28425 gse36004 and cse79181 [...] | custom analysis and heatmap generation code is available from https://githubcom/petercomb [...] | The authors declare no competing financial interests. | Funding Not applicable. | Trial registration ClinicalTrials.gov ID NCT00371540 |
| **PHYS/MATH** | all data [...] have been made publicly available [...] through the following dois: 10 5281/zenodo 3238621 [...] | it is opensource and freely available on github [ref] | Competing interests The authors declare that they have no competing interests. | This study was supported by L'Agence Nationale de la Recherche (ANR); reference: ANR-09-BLAN-0093-03. | - |
| **SOC SCI** | the data are available through dryad at the following link: https://doi org/10 5061/dryad 53t31 . | we include r code for step-by-step instructions for our analysis in the supplemental material [...] | Competing interests: The authors declare that they have no competing interest. | Funding The research reported was not externally funded. | Trial registration Australian New Zealand Clinical Trials Registry ACTRN12611000438954 |
