# Supplementary material for: Assessment of transparency indicators across the biomedical literature: How open is open?
Source: PLoS Biol. 2021 Mar 1;19(3):e3001107. doi: 10.1371/journal.pbio.3001107 (PMC7951980; doi:10.1371/journal.pbio.3001107)
Supplement: S10 Table — Reviewer concordance was very good between the 2 new reviewers (S.S. and D.G.C.I), as well as the 2 reviewers and the previous reviewer (J.D.W.). Appreciable deviations were only seen in the assessment of Novelty, presence of a Replication component, and Funding disclosures, the latter of which reached statistical significance (95% CI, 0%–18%). All 3 of these were manually re-extracted and adjudicated by both reviewers to ascertain that these discrepancies did not reflect systematic differences in extraction. (DOCX) [file pbio.3001107.s013.docx]

**S10 Table. Reviewer concordance.**

| **Indicator** |  | **J.D.W.** | **D.G.C.I.** | **S.S.** | **P-value** |
| --- | --- | --- | --- | --- | --- |
| **Data sharing** | **No** | 76 (80.0%) | 138 (83.6%) | 143 (77.7%) | 0.369 |
|  | **Yes** | 19 (20.0%) | 27 (16.4%) | 41 (22.3%) |  |
| **Code sharing** | **No** | 95 (100.0%) | 162 (98.2%) | 182 (98.9%) | 0.454 |
|  | **Yes** | 0 (0.0%) | 3 (1.8%) | 2 (1.1%) |  |
| **COI disclosure** | **No** | 51 (34.5%) | 69 (30.3%) | 89 (32.8%) | 0.673 |
|  | **Yes** | 97 (65.5%) | 159 (69.7%) | 182 (67.2%) |  |
| **Funding disclosure** | **No** | 45 (30.4%) | 65 (28.5%) | 82 (30.3%) | 0.880 |
|  | **Yes** | 103 (69.6%) | 163 (71.5%) | 189 (69.7%) |  |
| **Protocol registration** | **No** | 90 (94.7%) | 155 (93.9%) | 172 (93.5%) | 0.967 |
|  | **Yes** | 5 (5.3%) | 10 (6.1%) | 12 (6.5%) |  |
| **Novelty** | **No** | 31 (32.6%) | 74 (44.8%) | 100 (54.3%) | 0.002 |
|  | **Yes** | 64 (67.4%) | 91 (55.2%) | 84 (45.7%) |  |
| **Replication** | **No** | 80 (84.2%) | 151 (91.5%) | 165 (89.7%) | 0.194 |
|  | **Yes** | 15 (15.8%) | 14 (8.5%) | 19 (10.3%) |  |
