# Supplementary material for: Assessment of transparency indicators across the biomedical literature: How open is open?
Source: PLoS Biol. 2021 Mar 1;19(3):e3001107. doi: 10.1371/journal.pbio.3001107 (PMC7951980; doi:10.1371/journal.pbio.3001107)
Supplement: S1 Text — (DOCX) [file pbio.3001107.s014.docx]

## S1 Text. Novelty and replication.

Novelty and replication are rather ambiguous. We hereby attempt to clarify which phrases were deemed to imply novelty and which were deemed to imply replication.

### Novelty

Phrases or words deemed to clearly indicate that the authors imply novelty:

1. Mentions of words such as: new/novel/revolutionary/ground-breaking/ innovative/etc.
   1. e.g. PMID 28035953 (abstract): “Three novel strains of photosynthetic bacteria from the family Ectothiorhodospiraceae were isolated”
2. Mentions of phrases such as: for the first time/never done before/not previously evaluated/etc.
   1. e.g. PMID 25758201 (introduction): “We, for the first time, show the involvement of GFAP-expressing cells in ductular reaction in chemically induced liver cirrhosis and show that there is no direct EMT in terms of myofibroblast development.”
3. Mentions of phrases such as: currently unknown/speculative/overlooked/etc.
   1. e.g. PMID 27873074 (introduction): “Results regarding the association between adipokine levels and bone mineral density (BMD) have been inconsistent; the effects of sex, menopause, and central obesity remain unknown. We evaluated the association between serum leptin, adiponectin, and high-molecular-weight (HMW) adiponectin levels and BMD according to menopause and central obesity status in Korean women.”
4. Mentions of phrases such as: we discovered that x is associated to y/our data show a novel function
5. Mentions of phrases such as: we developed an apparatus/tool/method/etc. to study x
   1. e.g. PMID 27087363 (introduction): “[...] a disposable bag clamped to a reusable base (Figure 1-B) was developed to enhance washout while allowing for the complete elimination of air during in vitro testing.”

Phrases or words not deemed to *clearly* indicate that the authors imply novelty:

1. Mentions of phrases such as: it is still unclear/not fully understood/poorly understood/incompletely understood/remains elusive/only a few studies/little is known/limited knowledge/inconsistent findings/remains controversial/etc.
   1. e.g. PMID 29154423 (abstract): “Because of the effect of pre-load vs after-load on these mechanisms is not completely understood, we studied the effect in isolated muscle strips.”
   2. e.g. PMID 27633504 (abstract): “It is not fully understood where and how people are exposed to sensitizing metals. Much can be learnt from studying occupational settings where metals are handled.”
2. Mentions of phrases such as: provides further evidence/deepen our understanding/incremental knowledge/etc.
   1. e.g. PMID 26373460 (abstract): This study provides further evidence for the post-regression stability that characterises Rett syndrome. Emergent low mood in Rett syndrome requires further research.”
   2. e.g. PMID 29321952 (abstract): “Based on previous evidence suggesting a possible nuclear role for FEZ1, we wanted to deepen our understanding of this function by addressing the FEZ1-RAR interaction. We performed in vitro binding experiments and assessed the interface of interaction between both proteins.”

Phrases that depend on the context:

1. Mentions of phrases such as: extended this concept/extended this query/etc.
   1. e.g. PMID 25870189 (introduction) - implied novelty: “This method is proposed for assessing RhoA-Rho kinase activity in patients with hypertension and other cardiovascular diseases. In this study, we extended this concept to the analysis of GEF activity in human.”

### Replication

Phrases or words deemed to *clearly* indicate that the authors imply some kind of replication:

1. Mentions of words such as: replication/replicate/repeat/validate/confirm/etc.
   1. PMID 28587893 (introduction): “It is therefore an important model in which to validate the use of IF and provide baseline data on neuronal numbers and neuronal density.”
   2. PMID 25625488 (introduction): “The aim of this study was to confirm this association and to elucidate the effects of the variant on protein function and Alzheimer-type pathology.”
2. Mentions of phrases such as: examine the estimate/accuracy/reliability.
   1. PMID 29274866 (introduction): “However, limited data on NS5A-inhibitor treatment escape have been obtained in such systems. We aimed at providing an independent head-to-head comparison of the efficacy of all currently licensed HCV NS5A inhibitors against the major HCV genotypes and important subtypes, as well as resistant escape variants, in infectious culture systems.”
3. Mentions of phrases: long-term follow-up study/etc.
   1. PMID 20469608 (introduction): “The 18-year follow-up of the PIAF cohort was recently completed and airway responsiveness to histamine was assessed in all available subjects.”

Phrases or words not deemed to clearly indicate that the authors imply some kind of replication:

1. Mentions of phrases: little is known/poorly understood/not clear/debated/limited information/etc.
   1. PMID 30051367 (introduction): “While some reports have indicated that sarcopenia is not associated with poor outcomes in resectable GC, other reports have indicated that visceral and subcutaneous fat are equivocal prognostic factors. 16 Thus, there are no clear data regarding the prognostic value of preoperative body composition parameters in patients of resectable GC.”
2. Mentions of phrases: provide further evidence/further characterize/more data/data are scarce/information is limited/only one study/etc.
   1. PMID 26373460 (introduction): “The purpose here was to add to the evidence about age-related clinical and behavioural change into adulthood.”
3. Mentions of phrases: consistent with previous findings/etc.
   1. PMID 30030228 (abstract): “Consistent with previous publications, decreased membrane fluidity was associated with increased fatty acid production ability”

### Novelty and Replication

Several articles mention words/phrases that classify them into both categories:

1. Mentions of phrases such as: repeated/tested/validated in a new sample/population/condition.
   1. e.g. PMID 27570184 (introduction): “The majority of previous studies were conducted in Western settings hence studies that explore attentional bias and eating behaviors among dieters and nondieters from Eastern cultures could shed light on relevant cultural differences.”
   2. e.g. PMID 27477952 (introduction): “Although JHFRAT has been validated in western countries, it may not be suit-able to use on Chinese population. Nunnally and Bernstein (1994) have also stated that validation of measurements is an unending process and that the validity of each use within a specific context must be documented empirically. Therefore, this study tried to test the validity and reliability of JHFRAT for its implication in Chinese population.”
