# Supplementary material for: Assessment of transparency indicators across the biomedical literature: How open is open?
Source: PLoS Biol. 2021 Mar 1;19(3):e3001107. doi: 10.1371/journal.pbio.3001107 (PMC7951980; doi:10.1371/journal.pbio.3001107)
Supplement: S2 Text — (DOCX) [file pbio.3001107.s015.docx]

**S2 Text. Algorithm validation.**

## Data sharing

Our aim was to evaluate the performance of the data sharing identification algorithm by N.R. in a set of 6017 randomly identified articles of 2015-2019 from PubMed Central (PMC). Specifically, we were interested in the expected sensitivity, specificity, accuracy and AUROC of this algorithm for various definitions of data sharing in English research articles found on PMC. Note that by research articles we refer to any articles with empirical data and we do not include case studies, systematic reviews or meta-analyses.

In considering possible definitions of data sharing, we first define the following modes of data sharing: (a) upon request – a statement that data are being made available upon request, (b) effective – immediately accessible data (e.g. URL works, etc.), (c) active – new data made available online (a subset of effective), (d) per-paper - the paper refers to immediately accessible data (regardless of whether the URL works or not) (a superset of effective), (e) public – use of previously generated public data and (f) false claims – data sharing statements that make false claims (e.g. authors claim all data were made available but only some was made available, authors claim that data are made available in the text but only statistics are made available, etc.). It should be noted that the data sharing algorithm was designed to fulfil the definition of “active” data sharing and this is the definition being used in our automated assessment of the open access articles on PMC Open Access, as presented in the main manuscript.

On the basis of these, we constructed the following definitions of data sharing: (a) *any statement* sharing refers to any mention of openly available data or intent to share data (= upon request + effective sharing + false claims); (b) *effective* sharing refers to sharing any immediately available data (= any statement – upon request – false claims (no raw data, page not found)); (c) *active* sharing refers to effective data sharing of new data (= effective data sharing – public); (d) *per-paper* sharing refers to articles that, as far as we can tell from the paper alone, make at least some of their data immediately available (= effective data sharing + page not found (i.e. the subset of ineffective data sharing that we could tell is ineffective from the paper itself)); (e) *public* sharing refers to the availability of any sharing excluding that of public data (= any statement - public); (f) *upon request* refers to any data sharing excluding statements of upon request (= any statement – upon request). Note that if an article contained more than one type of data sharing, the most broadly applicable mode was selected (for example, if an article both made data available on GEO and also had a statement of data sharing upon request, we counted the former).

Performance was first assessed in 100/764 randomly identified articles that the algorithm predicted to share data. Within these data, two reviewers confirmed 100 research articles in English, of which 5 made a false claim (3 claimed to provide all data but only provided some data, 2 claimed to share data on an inaccessible URL and 1 claimed to share raw data where none could be found) and 4 used public data. As such, across different definitions, there were 100 true positives (TPs) for any data sharing, 97 TPs for effective data sharing, 93 TPs for active data sharing, 99 TPs for per-paper sharing, 96 for public data sharing and 100 for upon request sharing.


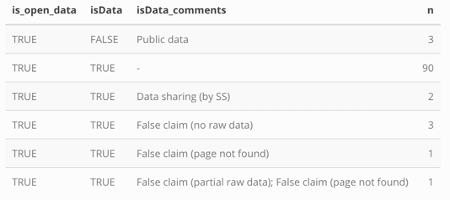


Performance was then assessed in 116/5253 randomly identified articles that the algorithm predicted to not share data. Within these articles, two reviewers confirmed 116 English articles, of which 89 were research articles; the three most common types of non-research articles were non-systematic reviews (6/27), case reports (6/27) and systematic reviews (4/27). Of the 89 research articles, 15 claimed at least some data sharing, of which 5 provided at least some effective data, 5 made false claims about data sharing (4 claimed raw data where none existed, 1 provided an inaccessible URL) and 1 at least partly used public data. This implies that there were 74 true negatives (TNs) for any data sharing, 83 TNs for effective data sharing, 84 TNs for active data sharing, 84 TNs for per-paper sharing, 75 TNs for public data and 78 TNs for upon request.


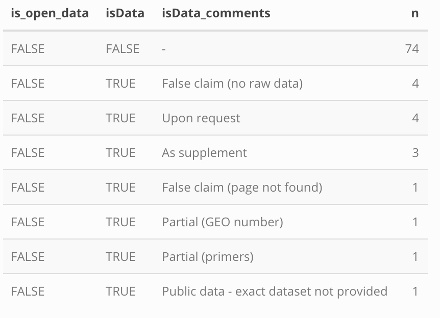


Of the 5 missed by per-paper data sharing, the GEO was missed because the tokenizer split the GEO from the GSE number (i.e. it split the database name from the dataset ID number), the primer was missed because the algorithm does not capture primers and the 3 supplements were missed because the algorithm does not capture “supplementary information”, “supplemental material” and only considers supplements that mention some kind of “supplemental table”. The algorithm could be improved by (a) fixing the tokenizer (do not split on all full stops, only those that symbolize next sentence) (fixes article 01665), (b) identifying .xlsx as referring to sharing a supplementary table (fixes article 00853) and (c) making the algorithm recognize S1 Table (as well as the currently recognizable Table S1) (fixes article 02339). The primer article (article 02236) is difficult to fix and the remaining article’s supplement could not have been recognized as true data sharing without access to the supplement (00856).

Overall, by extrapolating our sample to the full 6017 articles, we would expect the following performance.

| **Definition** | **TP** | **FP** | **TN** | **FN** | **Sens** | **Spec** | **Acc** | **AUROC** |
| --- | --- | --- | --- | --- | --- | --- | --- | --- |
| **Predicted** | **n = 764 research** | | **n = 4030 research** | | **-** | **-** | **-** | **-** |
| **Any statement** | 764 | 0 | 3351 | 679 | 52.9 % | 100 % | 85.8 % | 76.5 % |
| **Effective** | 741 | 23 | 3759 | 271 | 73.2 % | 99.4 % | 93.9 % | 86.3 % |
| **Active** | 711 | 53 | 3804 | 226 | 75.9 % | 98.6 % | 94.2 % | 87.3 % |
| **Per paper** | 756 | 8 | 3804 | 226 | 77.0 % | 99.8 % | 95.1 % | 88.4 % |
| **Public** | 733 | 31 | 3396 | 634 | 53.6 % | 99.1 % | 86.1 % | 76.4 % |
| **Upon request** | 764 | 0 | 3532 | 498 | 60.5 % | 100 % | 89.6 % | 80.3 % |

By correcting the GEO and fixing the two supplements, by the per-paper definition, we would get 756 TP, 8 FP, 3940 TN and 90 FN, thus, 89.4% sensitivity, 99.8% specificity, 98.0% accuracy and 94.6% AUROC.

### Summary

Upon manual inspection of a random sample of 100 research articles of 2015-2019 from PMC that were labelled as actively sharing data, 93 were indeed found to actively share data, but 7 were not - 4 used publicly available data, 2 referred to an inaccessible URL and 1 claimed that all raw data were in the text where none could be found. Similarly, out of 89 research articles labelled as not actively sharing data, 84 were found to not share data, but 5 did - 3 made their data available as supplements, 1 referred to a GSE number and 1 contained a primer sequence. Assuming similar proportions across all 6017 articles in our sample, in terms of active data sharing in research articles, this algorithm has an accuracy of 94.2% (95% CI, 89.7-97.99%), a sensitivity of 75.8% (95% CI, 61.4-93.9%) and a specificity of 98.6% (95% CI, 97.6-99.5%). Applying this algorithm across research articles of 2015-2019 on PMC is likely to underestimate the true proportion of data sharing by an absolute value of 3.6% (i.e. for every 4794 random PMC research articles of 2015-2019, we expect this algorithm to label 764 positive, whereas 935 are actually positive).

## Code sharing

Our aim was to evaluate the performance of the code sharing identification algorithm developed by N.R. in a set of 6017 randomly identified articles of 2015-2019 from PubMed Central (PMC). Specifically, we were interested in the expected sensitivity, specificity, accuracy and AUROC of this algorithm for various definitions of code sharing in English research articles found on PubMed Central. Note that by research articles we refer to any articles with empirical data and we do not include case studies, systematic reviews or meta-analyses.

In considering possible definitions of code sharing, we first defined the following modes of code sharing: (a) any – any reference to openly available code or intent to share code, (b) effective – immediately accessible code (e.g. URL works, etc.), (c) active – statement that new code is being made available online (a subset of effective), (d) per-paper - the paper states that the code is immediately accessible (regardless of whether the URL works or not) (a superset of effective), (e) public – use of previously written code (e.g. a public tool) and (f) false claims – code availability statements that make false claims (e.g. authors claim all code was made available but only some was made available, etc.).

On the basis of these, we constructed the following definitions of code sharing: (a) *any statement* sharing refers to any mention of openly available code or intent to share code (= any); (b) *effective* sharing refers to sharing any immediately available code (= any statement – false claims); (c) *active* sharing refers to effective sharing of new code (= effective – public); (d) *per-paper* refers to articles that, as far as we can tell from the paper alone (without the supplement or accessing the web), make at least some of their code immediately available (= effective + page not found (i.e. the subset of ineffective code sharing that we could tell is ineffective from the paper itself)); (e) *public* sharing refers to the availability of any code sharing excluding public code (= any - public). Note that if an article contained more than one type of code sharing, the most broadly applicable mode was selected (for example, if an article both used public code and made a false claim that all code was available, we counted only the public code).

Performance was first assessed in all 117 articles that the algorithm predicted to share code. Two reviewers confirmed 110 research articles in English, of which 7 did not mention any code sharing, 4 referred to at least some use of publicly available tools and 3 represented ineffective code sharing (inaccessible URL; one of these three also used a publicly available tool). This implies that there were 103 true positives (TPs) for any statement sharing, 101 TPs for effective sharing, 97 TPs for active sharing, 103 TPs for per-paper sharing and 99 TPs for public sharing.

Of the articles falsely predicted to share code across different definitions of code sharing, 3 included references that mentioned GitHub, 2 mentioned GitHub without providing code on GitHub (e.g. "this dataset is available on GitHub") and 2 mentioned words of the main text misconstrued to refer to code sharing (e.g. “coding sequence”). The 3 mentions of GitHub from the references and the mention of a coding sequence are aspects of this code that can be fixed more easily than the rest.


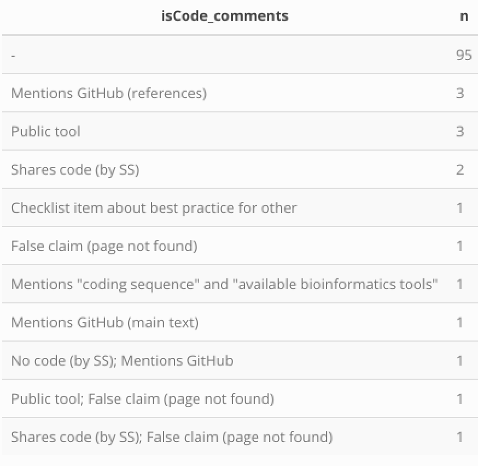


Performance of code sharing was also assessed in the sample of articles assessed for data sharing. Of 100/764 randomly identified articles that the algorithm predicted to share data and 116/5253 articles that the algorithm predicted to not share data, there were 189 research articles in English.

Of 100/100 research articles predicted to share data, 7 were also predicted to share code (i.e. 93 were predicted to not share code). Of 93 predicted to not share code, 89 were indeed true negatives (TNs), but 4 were false negatives (FNs). Of 4 FNs, one mentioned BitBucket, one mentioned R-syntax (instead of R script), one mentioned GitHub within a URL rather than its own word (the algorithm is looking for "\\bgithub\\b", which only recognizes the word GitHub) and one was missed because it made its code available on ResearchGate (albeit this was a false claim as the code was not found on ResearchGate upon looking). The algorithm could be updated to identify all 4 of these cases in terms of per-paper code sharing.

Of 89/116 research articles predicted to not share data, 88 were predicted to not share code (i.e. negative) and 1 was predicted to share code (i.e. positive). Of 88 predicted negative, 1 was a FN and shared its code as a supplement: “Our methods were easy to implement in R, and the code is presented in Supplementary Table 1”. There were no FPs. This could be fixed by identifying the combination of code with supplement, in a similar fashion to the identification of data in combination with supplement.

Performance metrics were calculated by extrapolating the observed performance across the expected number of research articles within all 6017 random articles. To calculate overall performance we used the articles predicted to share code to estimate TP/FP and the articles stratified by data sharing to estimate TN/FN. Given the association of data sharing with code sharing, this provided a more efficient approach to estimating performance and is formally known as importance or stratified sampling.

As an example, we hereby illustrate how this approach was used to calculate the performance metrics for Any statement. TP = 103; FP = 7; TN = 90/93 * 93/93 * 703 + 87/88 * 88/114 * 5197; FN = 3/93 * 93/93 * 703 + 1/88 * 88/114 * 5197. Notice that we are using 703 instead of 764 and 5197 instead of 5253 because we are only considering those predicted positive or negative for data sharing within the articles not sharing code (i.e. 6017 - 117 = 5900). Also notice that we are using 93 instead of 100 and 114 instead of 116 because we are only using observations that were predicted negative for code sharing - this can be done without complications because of random sampling (i.e. sampled positive are independent of sampled negative). Finally, notice that the total number of expected research articles is slightly different from that in data sharing (4825 vs 4794) because this was estimated using a different sample of the data; this uncertainty was taken into account when calculating the 95% confidence intervals presented in the main text.

| **Definition** | **TP** | **FP** | **TN** | **FN** | **Sens** | **Spec** | **Acc** | **AUROC** |
| --- | --- | --- | --- | --- | --- | --- | --- | --- |
| **Predicted** | **n = 110 research** | | **n = 4715 research** | | **-** | **-** | **-** | **-** |
| **Any statement** | 103 | 7 | 4639 | 76 | 57.5 % | 99.8 % | 98.3 % | 78.7 % |
| **Effective** | 101 | 9 | 4647 | 68 | 59.8 % | 99.8 % | 98.4 % | 79.8 % |
| **Active** | 97 | 13 | 4647 | 68 | 58.8 % | 99.7 % | 98.3 % | 79.3 % |
| **Per paper** | 103 | 7 | 4639 | 76 | 57.5 % | 99.8 % | 98.3 % | 78.7 % |
| **Public** | 99 | 11 | 4639 | 76 | 56.6 % | 99.8 % | 98.2 % | 78.2 % |

With the suggested updates to the algorithm (reference avoidance, “coding sequence” avoidance and inclusion of BitBucket, R-syntax, ResearchGate, www.github.com, and supplementary tables) the per-paper performance would been perfect (i.e. 100% for all performance metrics), even though this is an overestimate due to overfitting.

### Summary

Upon manual inspection of all 110 research articles of 2015-2019 from PMC that were labelled as actively sharing code, 97 indeed shared at least some code, whereas 4 did not – all 4 of these mentioned using code by publicly available code, e.g. “local realignment and variation call were analyzed using Samtools10 Picard (http://broadinstitute.github.io/picard/) and GATK”. Similarly, out of 181 articles labelled as not sharing code, 177 indeed did not share code, but 4 did. Three of these also shared data and uploaded their code on BitBucket, OSF or referred to it as R-syntax, all three of which were not identified by the algorithm. The fourth did not share data and was missed because it had made its code available as a supplement: “Our methods were easy to implement in R, and the code is presented in Supplementary Table 1, <http://links.lww.com/MD/B200>.” This final mistake had a major impact on the estimated sensitivity because the majority of articles did not share data (5253/6017) and as such this one article was dramatically overweighted. Assuming similar proportions across all estimated 4825 research articles, this algorithm has an accuracy of 98.3% (95% CI, 96.0-99.6%), a sensitivity of 58.7% (95% CI, 34.0-93.7%) and a specificity of 99.7% (95% CI, 99.6-99.9%). Applying this algorithm across the whole PMC is likely to underestimate the true proportion of code sharing by an absolute value of 1.1% (i.e. for every 4825 random PMC research articles of 2015-2019, we expect that this algorithm will label 110 as positive, whereas 164 are actually positive).

## Conflicts of interest

Our aim was to develop and evaluate a conflict of interest (COI) disclosure identification algorithm. The algorithm was developed in a set of 500 randomly identified articles from PubMed (2015-2018) and evaluated in a set of 6017 randomly identified articles from PubMed Central (2015-2019). Specifically, we were interested in the expected sensitivity, specificity, accuracy and AUROC of this algorithm for various definitions of COI disclosures found on PubMed Central.

We understand COI as arising “whenever activities or relationships compromise the loyalty or independent judgment of an individual who is obligated to serve a party or perform certain roles.” ([Rodwin, 2017](https://papers.ssrn.com/sol3/papers.cfm?abstract_id=3084307)). In building this algorithm, we operationalize this definition by identifying all occasions where authors clearly declare what they call “conflicts of interest” (and synonyms of that, e.g. “Competing interests”) and whenever authors clearly report evidence of financial gain (e.g. “J.K. receives fees/stock/benefits from GSK” or “J.K. is employed/on the advisory board of/a presenter for GSK”), but not when the financial gain is implied but not clearly stated (e.g. if an author states as their affiliation GSK, but does not clearly state “J.K. is employed by GSK.” What authors call “disclosures” or “financial disclosures” were only considered to represent COIs whenever they used language reminiscent of our working definition (e.g. “Financial disclosures: no competing interests” or “Financial disclosures: J.K. is a consultant for GSK” or “Financial disclosures: Nothing to disclose.”) and not otherwise (e.g. “Financial disclosures: J.K. received a grant by the NIH.”). We recognize that there are many types of interests (for example, semantically “conflicts of interest” is not identical to “competing financial interests”), that commercial financial gain may not always be a conflict of interest, that reporting a conflict of interest does not imply you do not have other unreported conflicts of interest. These are left for future exploration, which we aid by creating an algorithm that, in its output, denotes why each disclosure was considered a COI (e.g. “J.K. is a consultant for GSK” would be labelled as “consultant”).

We explored the performance of our algorithm across three definitions of performance: (a) performance across any article on PubMed Central, (b) performance across non-English articles, (c) performance across articles in which the PDF to text conversion was successful, (d) performance across articles with explicit vs non-explicit disclosures of COI (e.g. “The authors declare no conflicts of interest” vs “J.K. receives benefits from GSK.”), (e) performance across research articles vs non-research articles.

### Algorithm development

The 500 randomly identified articles from PubMed (2015-2018) were split into a train, validation and test set (7:1.5:1.5). We first developed an algorithm on the basis of the train and tested in the validation set. We then improved the algorithm on the basis of the validation set and tested in the test set. We then improved the algorithm on the basis of the test set and tested in the 6017 PubMed Central articles. This structure allowed us to appreciate the performance of our algorithm during development to understand whether the approach we were following was appropriate and whether the algorithm was likely to perform well in the final test set; by using pre-randomized dataset cuts, we avoided biases inherent to stopping rules.

Upon testing in the 6017 PubMed Central articles, we understood that our algorithm was significantly underperforming in recognizing COI disclosures using non-standard language (e.g. “J.K. received benefits from GSK.”). Such disclosures were not encountered during training, which is why the algorithm was not built to capture them – upon rescreening the articles, we identified that such disclosures had been made on some occasions, but not identified by the screening team.

In testing within the 6017 articles, the initial algorithm identified 1225 articles that it predicted did not share any COIs and 4792 that did. We took the 1225 articles and split them again into 3 groups: a train group (500, including 180 in which we tested the initial algorithm), a validation group (500) and a test group (225). The test group was only used when algorithm development was complete and this is the performance that we report here – the algorithm has now been modified in view of the mistakes seen in this test set, but not re-evaluated, so we understand this performance as the minimum possible in the target population.

### Algorithm assessment

As indicated above, the initial algorithm was used to test 6017 articles. We then went through a sub-sample of 100 articles predicted to share a COI disclosure and 180 articles predicted to not share a COI disclosure. These numbers were calculated on the basis of the initial assessment, which predicted no false positives and 3 false negatives.

As indicated above, a second algorithm was developed to cater for non-standard COI disclosures. This was trained and assessed in the 1225 articles that the first algorithm predicted negative (trained in 1000, tested in 225). We then went through all 225 test articles to evaluate the performance of the algorithm.

### Initial development

The train had 356 articles, 339 of which were in English (this is quite different from the PubMed Central, which appears to have about 3 non-English articles per 200). The algorithm had 100% sensitivity and specificity by design in this set. Of 78 articles in the validation set, 70 were in English. There was only 1 false negative (FN) out of 41 known positives because of an unsuccessful conversion from PDF to text. The test set had 78 articles, 76 of which in English. In this set, the algorithm had 100% specificity. Of the 55 known positives, there were 3 FNs: one because of an unsuccessful conversion to text, one because of a spelling mistake in the text (“confict” instead of “conflict”) and one because the algorithm did not identify a true disclosure of COI, even though it should have. In the process, the algorithm also identified 8 occasions where the reviewers failed to identify the COI disclosure (the reviewers had 7 FNs and 1 FP).

### Initial evaluation

Given that there were 0 false positives (FPs) and 1/55 FNs in the test set during initial algorithm development and that we want to have an estimate within a margin of error of 2% (e.g. 97%, 95-99%), using the formula sqrt((1/55) * (54/55)/180) * 2 = 0.02, we used 100 articles that the algorithm predicted positive and 180 articles predicted negative.

In the test set of 100 FPs, all 100 were true positives (TPs). Of the 180 predicted negative, 136 were deemed research articles and 4 were non-English articles (2 French, 1 Italian, 1 Chinese); none of the non-English articles was non-research. Out of 180 predicted negative, there were 172 TN and 8 FN: 2 were in French, 1 was an unsuccessful conversion from PDF to text (if this were successful, the algorithm would have labelled it correctly) and 5 did not use the standard language observed in the sample of 500 articles on the basis of which this algorithm had been developed; 4/5 of these articles declared conflicts with the industry and 1/5 declared that their study was not commercially sponsored. The following table presents performance across definitions:

| **Definition** | **Sensitivity** | **Specificity** | **Accuracy** |
| --- | --- | --- | --- |
| **Any disclosures** | 97.5% | 100% | 98% |
| **English disclosures** | 97.8% | 100% | 98.2% |
| **Well-converted English disclosures** | 97.9% | 100% | 98.3% |
| **Explicit disclosures** | 99.6% | - | - |
| **Non-explicit disclosures** | 0.0% | - | - |
| **Any disclosures in research** | 97.6% | 100% | 98.0% |
| **Any disclosures in non-research** | 97.2% | 100% | 98.0% |

### Subsequent development

The train had 500 articles previously predicted to not have a COI disclosure. Using an updated version of the algorithm according to performance in the previous test (the data of which were included in this train), 29 positives were predicted (5.8%). Out of 18 that had not been seen before, 6 were true positives (3 no involvement of funder disclosures, 2 in French, 1 employee of organization disclosure). The remaining 12 were FPs (67%) (6 because of "relationship", 1 because of "connection", 1 because of "employee", 1 because of "consulting", 3 unknown). One of the articles that was previously part of the test was also a FP. There were 8/146 assessed predicted negative that were FN (5%).

The algorithm was improved by creating a module designed to look for all of these non-explicit disclosures of conflict, as well as disclosures like “This study was not commercially sponsored.”, within the text typically found between Funding/Acknowledgement sections and the Reference section. It was then re-assessed in the validation set of another 500 articles that were previously deemed negative. Out of 500, 30 (6%) were deemed positive. Out of 30 predicted positive, 26 were TP, but 4 were FP (i.e. 13% of positive are false positive). This is a substantial improvement from the previous algorithm (from 67% to 13%), but worse than the initial algorithm, which had no false positives. 2/4 mistakes occurred because the algorithm was not constrained to the Acknowledgements part due to non-standard language to denote Acknowledgements. Out of 250 predicted negative that were assessed, 16 were FN (6.4%). This remains within the same range as the FNs in the training set (5%), which suggests that many of the improvements simply overfitted to the train data, rather than meaningfully improving the algorithm (in terms of improving sensitivity).

On the basis of these results, the algorithm was improved to be better restricted to Acknowledgements, identify more words related to non-explicit COI disclosures (e.g. “honoraria”, “advisory board”, “commercial relationship”) and certain commonly seen phrases, such as “The funding sources of this study had no role in study design.” It was then reassessed in the final an unseen test set of 225 previously deemed negative studies.

### Subsequent evaluation

Out of 225 previously deemed negative, 176 (78.2%) were research articles in English. Of the remaining 49, 3 (1.3%) were not in English (2 Chinese, 1 Spanish) and 46 were not research articles (11.6%). One of the 3 articles not in English (the one in Spanish) had a disclosure for conflicts of interest. For all articles (including non-English articles), there were 7 FN (out of 216 deemed negative, 3.2%) and 1 FP (out of 8 deemed positive, 12.5%). Both improvement in FNs (5%, then 6.5%, now 3.2%) and FPs (67%, then 13%, now 12.5%) was substantial, but the improvement in FPs from the validation to test was negligible. This suggests that, despite the improvements in the algorithm on the basis of its performance in the test set, we were unlikely to see substantial improvement in subsequent validation attempts. This is the assessment of the updated algorithm across definitions of COI:

| **Definition** | **Sensitivity** | **Specificity** | **Accuracy** |
| --- | --- | --- | --- |
| **Any disclosures** | 99.2% | 100% | 99.3% |
| **English disclosures** | 99.3% | 100% | 99.4% |
| **Well-converted English disclosures** | 99.4% | 100% | 99.5% |
| **Explicit disclosures** | 99.9% | - | - |
| **Non-explicit disclosures** | 55.6% | - | - |
| **Any disclosures in research** | 99.3% | 99.4% | 99.4% |
| **Any disclosures in non-research** | 97.2% | 100% | 98.0% |

### Current algorithm

The absolute final algorithm was used to once again assess the very first test (the one with 180 articles), in which it successfully recognized 7 COI disclosures that the reviewer had not previously seen, raising the number of FNs from 11 to 18. In other words, within articles that were predicted negative by the initial algorithm, the final algorithm increased the number of COI disclosures recognized by 64% over the human reviewer.

### Summary

Upon manual inspection of 100 articles labelled positive for COI, all 100 indeed reported a COI disclosure. Similarly, out of 225 labelled negative, 218 were indeed negative, but 7 were positive. Of these 7, 1 was in Spanish, 1 was an unsuccessful conversion of PDF to text (had it been successful, it would have been identified), and 4 of the remaining 5 used non-standard language to describe COIs (e.g. “No benefits in any form have been received or will be received from a commercial party.”). Running the COI algorithm within the sample of 499 articles from PubMed, we identified 7 articles that had previously been missed by the two reviewers. Assuming similar proportions across all 6017 articles, our algorithm has an accuracy of 99.3% (95% CI, 98.8-99.7%), a sensitivity of 99.2% (95% CI, 98.6-99.7%) and a specificity of 99.5% (95% CI, 98.5-100.0%). Applying this algorithm across the whole PMCOA is likely to underestimate the true proportion of COIs by an absolute value of 0.5% (i.e. for every 6017 random PMCOA articles, we expect this algorithm to label 4840 vs 4873 positive).

## Funding

Our aim was to develop and evaluate a funding disclosure identification algorithm. The algorithm was developed in a set of 500 randomly identified articles from PubMed (2015-2018) and evaluated in a set of 6017 randomly identified articles from PubMed Central (2015-2019). Specifically, we were interested in the expected sensitivity, specificity, accuracy and AUROC of this algorithm for various definitions of funding disclosures found on PubMed Central.

### Funding definitions

We understand a funding disclosure as an explicit declaration of all sources from which funds were used in completing a study. In building this algorithm, we operationalized this definition by identifying any explicit mentions of funding or financial support. Some of these mentions were found within their own paragraphs, some within Acknowledgements, some within footnotes, some at the very start of a publication. We purposefully avoided mentions of financial relationships or financial disclosures more akin to a COI disclosure, e.g. “Financial disclosures: Nothing to disclose.” This is a very unclear statement to us. We purposefully also avoided statements such as: “Information on authorship, contributions, and financial & other disclosures was provided by the authors and is available with the online version of this article at www.haematologica.org.” We also understood mentions such as “J.F. has received grant money from GSK” as COI disclosures and classified them like so. Our algorithm does not guarantee that the funding disclosure is true or that it is complete. Ambiguous statements like “We received support from XXX” were interpreted as a funding disclosure from some kind of foundation (e.g. NIH) and not as a funding disclosure if XXX was not a foundation (e.g. Mark Johnson).

We explored the performance of our algorithm across a few definitions of performance: (a) performance across any article on PubMed Central, (b) performance across non-English articles, (c) performance across articles in which the PDF to text conversion was successful, (d) performance across articles with explicit vs non-explicit disclosures of funding (e.g. “Funding: The authors received funding from NIH” vs “Acknowledgements: The authors thank the Wellcome Trust for its support.”), (e) performance across research articles vs non-research articles.

### Algorithm development

The 500 randomly identified articles from PubMed (2015-2018) were split into a train, validation and test set (7:1.5:1.5). We first developed an algorithm on the basis of the train and tested in the validation set. We then improved the algorithm on the basis of the validation set and tested it in the test set. We then improved the algorithm on the basis of the test set and tested in the 6017 PubMed Central articles. This structure allowed us to appreciate the performance of our algorithm during development to understand whether the approach we were following was appropriate and whether the algorithm was likely to perform well in the final test set; by using pre-randomized dataset cuts, we avoided biases inherent to stopping rules.

Upon testing in the 6017 PubMed Central articles, we understood that our algorithm was significantly underperforming in recognizing funding disclosures using non-standard language (e.g. “The authors thank the Wellcome Trust for its support.”). Such disclosures were not encountered during training, which is why the algorithm was not built to capture them.

In testing within the 6017 articles, the initial algorithm identified 995 articles that it predicted do not share any funding disclosures and 5022 that did. We took the 995 articles, saved 225 as a test and then tested the algorithm in batches of 50. Every time, we tested in the next 50, then developed the algorithm to correctly identify those 50 and then tested in the text 50, and so on. The rule was to stop when performance appears to remain stable across batches of 50. The test group was only touched when algorithm development was complete and this is the performance that we report – note that the algorithm has now been modified in view of the mistakes seen in this test set, but not re-evaluated, so we understand this performance as the minimum possible in the target population.

### Algorithm assessment

As indicated above, the initial algorithm was used to test 6017 articles. We then went through a sub-sample of 100 articles predicted to share a funding disclosure and 100 articles predicted to not share a funding disclosure. These numbers were arbitrary as we knew that we would be redeveloping the algorithm – we merely wanted an informally representative enough sample based on our experience while developing.

As indicated above, a second algorithm was developed to cater for non-standard funding disclosures. This was trained and assessed in the 995 articles that the first algorithm predicted negative (trained in 769, tested in 226). We then went through all 226 test articles to evaluate the performance of the algorithm.

### Initial development

The train had 356 articles, 339 of which were in English (this is quite different from the PubMed Central, which appears to have about 3 non-English articles per 200). The algorithm had 100% sensitivity and specificity by design in this set. Of 77 articles in the validation set, there were 5 false negatives (FN) out of 51 known positives and 1 false positive (FP) out of 24 known negatives (Accuracy, 92.2%). Of 78 articles in the test set, there were 2 FNs out of 53 known positives and 4 FPs out of 25 known negatives (Accuracy, 92.3%). In the process, the algorithm also identified 9 occasions where the reviewers made an error (7 FN, 2 FP).

### Initial evaluation

In the test set of 100 FPs, all 100 were true positives (TPs). Of the 100 predicted negative, 50 were deemed research articles (i.e. provided empirical data or a new method) and 2 were non-English articles (1 French, 1 Chinese); none of the non-English articles was non-research. Out of 100 predicted negative, there were 88 TN and 12 FN, 1 of which errors was due to an unsuccessful conversion from PDF to text (if this were successful, the algorithm would have labelled it correctly) and 8 did not use the standard language observed in the sample of 500 articles on the basis of which this algorithm had been developed; 9 of the missed articles declared some kind of funding and 3 declared no funding. The following table presents performance across definitions:

| **Definition** | **Sensitivity** | **Specificity** | **Accuracy** |
| --- | --- | --- | --- |
| **Any disclosures** | 97.7% | 100% | 98.0% |
| **English disclosures** | 97.7% | 100% | 98.0% |
| **Well-converted English disclosures** | 97.9% | 100% | 98.2% |
| **Explicit disclosures** | 99.2% | - | - |
| **Non-explicit disclosures** | 00.0% | - | - |
| **Any disclosures in research** | 97.9% | 100% | 98.1% |
| **Any disclosures in non-research** | 94.6% | 100% | 97.3% |

### Subsequent development

Development proceeded to consider 450 articles in detail and another 319 for just false positives (i.e. only the articles that the algorithm said were positive were examined). The algorithm was improved with more types of funding titles and by trying to capture more non-explicit mentions of funding. Accuracy for successfully converted PDFs changed like so: 86% (1:50), 92% (51:100), 98% (101:150), 90% (151:200), 96% (201:250), 88% (251:300), 96% (201:350), 90% (351:400), 98% (401:450) and no false positives in 451:769 – the mean accuracy was 93.7%, excluding the first 100.

### Subsequent evaluation

Out of 226 previously deemed negative, 116 (51.3%) were research articles in English. Of the remaining 110, 1 (0.9%) was not in English (1 French) and did not have a funding disclosure. For all articles (including non-English articles), there were 4 FN (out of 211 predicted negative, 1.9%) and 4 FP (out of 15 predicted positive, 26.7%). As such, there was a substantial decrease in FNs (from 12% to 1.9%), but also, within this set a substantial increase in FPs (from 0% to 26.7%) – had these articles been assessed using the old algorithm, the accuracy would have dropped from 96.5% (218/226) to 93.4% (211/226). This suggested that the algorithm was better able to now identify non-standard mentions of funding, but at the cost of a loss in specificity.

| **Definition** | **Sensitivity** | **Specificity** | **Accuracy** |
| --- | --- | --- | --- |
| **Any disclosures** | 99.7% | 98.1% | 99.4% |
| **English disclosures** | 99.7% | 98.1% | 99.4% |
| **Well-converted English disclosures** | 99.7% | 98.1% | 99.5% |
| **Explicit disclosures** | 99.7% | - | - |
| **Non-explicit disclosures** | 100% | - | - |
| **Any disclosures in research** | 99.7% | 99.0% | 99.7% |
| **Any disclosures in non-research** | 100% | 97.2% | 98.4% |

Had we been using the old algorithm in this test set, the performance would have been: 98.7% sensitivity, 100% specificity, 100% PPV, 93.4% NPV and 98.9% accuracy for any disclosure.

### Current algorithm

The final algorithm was used to once again assess the very first test (the one with 100 articles), in which no more funding disclosures were recognized. This suggests that human performance is superior to machine performance. It should also be noted that adjudication of the 226 occurred before learning the response of the algorithm, which clearly indicates that human performance is superior.

### Summary

Upon manual inspection of 100 articles labelled positive for Funding, all 100 indeed had an explicit Funding disclosure. The algorithm was then calibrated to PMC articles that were initially labelled negative and then tested in a random unseen sample. Of 226 test articles initially labelled negative, the algorithm correctly predicted 218. Of the remaining 8, 4 were falsely predicted negative (FN) and 4 false predicted positive (FP). Of 4 FNs, 1 was an unsuccessful conversion of PDF to text (had it been successful, it would have been predicted positive) and 3 used uncommon language (e.g. “We would like to thank the U.S. Embassy in Addis Ababa, Ethiopia, Jigjiga University, and Texas Tech University for funding this research”). Of 4 FPs, 2 referred to funds received by another study and 2 contained words commonly seen in funding disclosures (“support” and “scholar”). Running the Funding algorithm within the sample of 520 articles from PubMed, we identified 7 articles previously mislabelled negative and 2 previously mislabelled positive. Assuming similar proportions across all 6017 articles, our algorithm has an accuracy of 99.4% (95% CI, 99.0-99.8%), a sensitivity of 99.7% (95% CI, 99.3-99.9%) and a specificity of 98.1% (95% CI, 96.1-99.5%). Applying this algorithm across the whole PMCOA is expected to identify the true proportion of funding disclosures (i.e. for every 6017 random PMCOA articles, we expect this algorithm to label 5084 vs 5084 positive). This algorithm can be improved in the future by (a) using a probabilistic model (e.g. a random forest) to predict outcome on the basis of the exported features and (b) by improving the quality of the extracted text from the PDFs.

## Protocol registration

Our aim was to develop and evaluate a registration statement identification algorithm. The algorithm was developed in a set of 500 randomly identified articles from PubMed (2015-2018) and evaluated in a set of 6017 randomly identified articles from PubMed Central (2015-2019). Specifically, we were interested in the expected sensitivity, specificity, accuracy and AUROC of this algorithm for various definitions of registration statements found on PubMed Central.

### Registration definitions

We understand a registration statement as any explicit declaration of study registration. This can include registrations on known websites, such as registration on ClinicalTrials.gov, as well as a previous publication of the protocol, as long as these registrations/protocols refer to the current study. Registered protocols that are not publicly available, such as statements like “The study was approved by the Ruakura Animal Ethics Committee (protocol no. 13899)” or “Alliance for Clinical Trials in Oncology (formerly Cancer and Leukemia Group B) Protocol #369901”, were not considered as protocol registration statements because they do not enhance transparency. Even though the algorithm was set up to also identify mentions such as “This study was not registered on any registry”, this ability is turned off by default because this statement does not enhance transparency in any way (i.e. it is not informative).

We explored the performance of our algorithm across a few definitions of performance: (a) performance across any article on PubMed Central, (b) performance across articles with explicit vs non-explicit statements of registration (e.g. “This study was registered on ClinicalTrials.gov with registration number NCT01234567”), (c) performance across research articles vs non-research articles and (d) performance within articles that mention NCT.

Note that by explicit we refer to any mentions of registration as a title, e.g. “Trial Registration: NCT…” or “Trial Number: NCT…” but not “Clinical Trial information: NCT…”, as clear registration statement, e.g. “Clinical Trial Registration: NCT…”, or as a clear phrase, e.g. “This study was registered on PROPSERO with ID number (…)” – for a phrase to be clear, it had to mention “registration” somewhere, in addition with the ID.

### Algorithm development

The 500 randomly identified articles from PubMed (2015-2018) were split into a train, validation and test set (7:1.5:1.5). We first developed an algorithm on the basis of the train and tested in the validation set. We then improved the algorithm on the basis of the validation set and tested it in the test set. We then improved the algorithm on the basis of the test set and tested in the 6017 PubMed Central articles. This structure allowed us to appreciate the performance of our algorithm during development to understand whether the approach we were following was appropriate and whether the algorithm was likely to perform well in the final test set; by using pre-randomized dataset cuts, we avoided biases inherent to stopping rules.

Upon testing in the 6017 PubMed Central articles, we understood that our algorithm was significantly underperforming in terms of false positives because of identifying mentions within references, mentions of NCT registrations within the introduction, mentions of NCT references as “this trial is underway” or in referring to the data being used, e.g. “We are using data from this trial (NCT01234567)”. This is why we redeveloped the algorithm in the first 100 articles and then tested the algorithm in the next 161 articles, out of 261 deemed positive by the initial algorithm.

In terms of false negatives, the initial algorithm had excellent performance. However, the re-developed algorithm falsely identified mentions of “registry” as evidence of a registration statement, many of which were not. This was fixed, for which reason we expect on average the reported test performance to be an underestimation.

### Algorithm assessment

As indicated above, the initial algorithm was used to test 6017 articles. We then went through 100 articles of predicted positives to appreciate algorithm performance and then assessed both the initial and redeveloped algorithm in the remaining 161 positive articles. Out of 5756 predicted negative, we used a stratified/importance sampling procedure by sampling 10/3248 articles deemed irrelevant (i.e. do not mention the words regist*/trial/NCT), 20/452 that were deemed relevant, 55/1962 out of those that were deemed relevant and had a Methods sections, 61/94 that contained an NCT identification number.

### Initial development

The train had 356 articles, 339 of which were in English. Developing this algorithm was not trivial because we had to compromise in what phrases were identified to balance sensitivity with specificity; given the very small number of positive examples in our train set, this was challenging. After initial development, the algorithm ran in 7 seconds in the train set with 4 FN (one of which because the line was inappropriately split) and 0 FPs (98.9% accuracy). The algorithm was corrected on the basis of these and was then re-tested in the 77 cases of the validation set, where there were eventually 76 TNs and 1 FN (the algorithm did not identify PROSPERO registration) (98.7% accuracy). This was corrected and the algorithm was then re-tested in the 78 records of the test set, where there were 73 TNs and 5 FNs (93.6% accuracy).

Eventually, using the latest iteration of the algorithm, we discovered another 3 TPs that had been missed by the reviewers and 1 TN, that had been labelled as positive by the reviewers.

### Initial evaluation

In the test set of 261 articles predicted positive, we randomly identified 161 as a test set. Of the 161 predicted positive, there were 147 TPs and 14 FPs (i.e. there were 14 errors). Most mistakes occurred because of articles referring to clinical trials underway, using the data of previously completed clinical trials or registration of their protocol with an IRB committee. Of the articles predicted negative, the following performance was observed (non-relevant articles, 21/21 TNs; relevant articles with no methods or NCT, 10/10 TN; relevant articles with methods and no NCT, 53/55 TN and 2/55 FN; articles referring to NCT, 55/61 TNs, 6/61 FNs). The following table presents performance across definitions when adjusted for the weighted sampling:

| **Definition** | **Sensitivity** | **Specificity** | **Accuracy** |
| --- | --- | --- | --- |
| **Any statements** | 97.3% | 99.6% | 99.5% |
| **Explicit statements** | 99.6% | - | - |
| **Non-explicit statements** | 69.3% | - | - |
| **Any statements in research** | 97.9% | 99.5% | 99.5% |
| **Any statements in non-research** | 91.9% | 100% | 99.8% |

### Subsequent development

Subsequent development occurred in 100/261 articles predicted positive. Given the small sample size, no assessment across collections of 50 articles was done. Additionally, no development in the articles predicted negative was done. Overall, given the performance in the test set, the development did not particularly improve performance, even though it substantially improved ability to identify non-explicit statements.

### Subsequent evaluation

Out of 161 articles previously predicted positive, the new algorithm identified 21 N and 140 P. Of these there were 147 TP, 4 FP, 10 TN, 5 FN (i.e. there were 9/161 errors). Of the 5756 articles predicted negative, the new algorithm predicted that 15 were in fact positive – of those there were 4 TP and 11 FPs. This suggests that the new algorithm is better in terms of specificity at the expense of sensitivity.

| **Definition** | **Sensitivity** | **Specificity** | **Accuracy** |
| --- | --- | --- | --- |
| **Any statements** | 95.6% | 99.7% | 99.5% |
| **Explicit statements** | 96.4% | - | - |
| **Non-explicit statements** | 85.4% | - | - |
| **Any statements in research** | 95.1% | 99.7% | 99.5% |
| **Any statements in non-research** | 100% | 99.8% | 99.8% |

### Current algorithm

The final algorithm was used to once again assess the very first test, identifying 3 previously non-identified statements as indicated above.

### Summary

Upon manual inspection of 161/261 articles labelled positive for a protocol registration statement, we found 5 FNs and 4 FPs. Of 5 FNs, 4 were grammatical failures of the algorithm to understand that the registration statement referred to the current and not some other study (e.g. "This registered study on www.clinicaltrials.gov (NCT01375270) was approved ...") and 1 was a statement contained within financial disclosures. Of 4 FPs, 2 were mentions of registration in the references, 1 referred to the registration of a study of which the data it was using and 1 was using a registered study as an example. Similarly, of 147/5657 articles initially labelled negative, there were 11 FPs and 2 FNs - note that the large number of errors occurred because of sampling from articles in which the algorithm was more likely to underperform (see Methods). Of 11 FPs, most errors occurred because of registrations that did not refer to open protocol registrations (e.g. approval by a medical ethics committee) and because of referral to other registries (e.g. a patient registry). Of the 2 FNs, 1 was a grammatical failure of the algorithm to understand that the registration statement referred to this study and 1 did not mention anything about registration, other than the NCT number (“EDITION 2 (NCT01499095) was a randomized, 6-month, multicenter, open-label, two-arm, phase IIIa study investigating ….”). Running the Registration algorithm within the sample of 499 articles from PubMed, we identified 2 positive and 1 negative studies that were previously erroneously labelled. Assuming similar proportions across all 6017 articles, our algorithm has an accuracy of 99.5% (95% CI, 99.3-99.7%), a sensitivity of 95.6% (95% CI, 92.0-98.6%) and a specificity of 99.7% (95% CI, 99.5-99.8%). Applying this algorithm across the whole PMCOA is likely to underestimate the true proportion of protocol registration statements by an absolute value of -0.14% (i.e. for every 6017 random PMCOA articles, we expect this algorithm to label 249 vs 241 positive).
