# Supplementary material for: Assessment of transparency indicators across the biomedical literature: How open is open?
Source: PLoS Biol. 2021 Mar 1;19(3):e3001107. doi: 10.1371/journal.pbio.3001107 (PMC7951980; doi:10.1371/journal.pbio.3001107)
Supplement: S3 Text — (DOCX) [file pbio.3001107.s016.docx]

## S3 Text. Associations of transparency with article characteristics.

Considering only publications from 2000 onwards, publications with a Conflict of Interest (COI) disclosure had substantially more references (Median, 38 vs 22), more often represented research articles (Proportion, 90.0% vs 85.3%) and tended to be published in journals of lower impact factor (Median, 2.8 vs 3.4) (Supporting Information Table 5). Publications with funding disclosures were primarily found in research articles (Proportion, 91.92% vs 78.7%) and had a median of more references (40 vs 18), more affiliations (3 vs 2), more authors (6 vs 4) and more citations (6 vs 4). Publications with registration statements had a median of more affiliations (4 vs 3), more authors (7 vs 5) and more tables (3 vs 1).
