# Supplementary material for: Assessment of transparency indicators across the biomedical literature: How open is open?
Source: PLoS Biol. 2021 Mar 1;19(3):e3001107. doi: 10.1371/journal.pbio.3001107 (PMC7951980; doi:10.1371/journal.pbio.3001107)
Supplement: S4 Text — (DOCX) [file pbio.3001107.s017.docx]

## S4 Text. Performance metrics.

We hereby define the performance metrics shown in Figure 2 and illustrate how they were calculated. The code for this procedure has been made available (see Code Availability). The performance metrics and their calculation are illustrated by using the test data for COI disclosures. The initial COI disclosures algorithm predicted that out of 6,017 random articles from PMC, 1,225 do not share COI disclosures and 4,792 do.

First, we took a random subsample of 100/4,792 articles predicted positive (i.e. a COI disclosure was found). We found:

|  | | **Manual assessment** | |  |
| --- | --- | --- | --- | --- |
|  |  | **True** | **False** |  |
| **Automated assessment** | **True** | True positive (TP) =  100 | False positive (FP) =  0 | PPV =  100 / (100+0) = 100% |
|  | **False** | False negative (FN) =  0 | True negative (TN) =  0 | NPV =  Not applicable |
|  | | Sensitivity =  100 / (100+0) = 100% | Specificity =  Not applicable | Accuracy =  100 / 100 = 100% |

*PPV = Positive Predictive Value (Precision). NPV = Negative Predictive Value.*

Prevalence (true) = (TP+FN) / (TP+FP+FN+TN) = 100/100 = 100%

Prevalence (estimated) = (TP+FP) / (TP+FP+FN+TN) = 100/100 = 100%

Error = Prevalence (true) - Prevalence (estimated) = 100% - 100% = 0%

Second, we took a random subsample of 225/1,225 initially predicted negative (i.e. a COI disclosure was not found). We found:

|  | | **Manual assessment** | |  |
| --- | --- | --- | --- | --- |
|  |  | **True** | **False** |  |
| **Automated assessment** | **True** | True positive (TP) =  8 | False positive (FP) =  1 | PPV =  8 / (8+1) = 89% |
|  | **False** | False negative (FN) =  7 | True negative (TN) =  209 | NPV =  209 / (7+209) = 97% |
|  | | Sensitivity =  8 / (8+7) = 53% | Specificity =  209 / (1+209) = 100% | Accuracy =  (8+209) / 225 = 96% |

PPV = Positive Predictive Value (Precision). NPV = Negative Predictive Value.

Prevalence (true) = (TP+FN) / (TP+FP+FN+TN) = 15/225 = 7%

Prevalence (estimated) = (TP+FP) / (TP+FP+FN+TN) = 9/225 = 4%

Error = Prevalence (true) - Prevalence (estimated) = 7% - 4% = 3%

Finally, we weighted the two tables into the statistics presented in Figure 2. This may be done by using the law of total probability with Bayes’ theorem. However, the same end-result may be achieved by using the aforementioned tables to estimate the expected table, had we manually assessed all 6,017 articles. Note that these numbers represent statistics of expected counts, not true counts:

|  | | **Manual assessment (expected counts)** | |  |
| --- | --- | --- | --- | --- |
|  |  | **True** | **False** |  |
| **Automated assessment (expected counts)** | **True** | TP =  100/100 * 4792 +  8/225 * 1225 = 4836 | FP =  0/100 * 4792 +  1/22 * 1225 = 56 | PPV =  4836 / (4836+56) = 99% |
|  | **False** | FN =  0/100 * 4792 +  7/225 * 1225 = 38 | TN =  0/100 * 4792 +  209/225 * 1225 = 1138 | NPV =  1138 / (1138+38) = 97% |
|  | | Sensitivity =  4836 / (4836+38) = 99% | Specificity =  1138 / (1138+56) = 95% | Accuracy =  (4836+1138) / 6017 = 99% |

*PPV = Positive Predictive Value (Precision). NPV = Negative Predictive Value.*

Prevalence (true) = (TP+FN) / (TP+FP+FN+TN) = (4836+38) / 6017 = 81%

Prevalence (estimated) = (TP+FP) / (TP+FP+FN+TN) = (4836+56) / 6017 = 81%

Error = Prevalence (true) - Prevalence (estimated) = 81% - 81% = 0%
