# Supplementary material for: Assessment of transparency indicators across the biomedical literature: How open is open?
Source: PLoS Biol. 2021 Mar 1;19(3):e3001107. doi: 10.1371/journal.pbio.3001107 (PMC7951980; doi:10.1371/journal.pbio.3001107)
Supplement: Attachment — Submitted filename: response-to-reviewers.docx [file pbio.3001107.s018.docx]

Response to reviewers

# DATA POLICY

You may be aware of the PLOS Data Policy, which requires that all data be made available without restriction: http://journals.plos.org/plosbiology/s/data-availability. For more information, please also see this editorial: http://dx.doi.org/10.1371/journal.pbio.1001797

Many thanks for depositing the raw data and code in OSF. However, we also ask that all individual quantitative observations that underlie the data summarized in the figures and results of your paper be made available in one of the following forms:

1. Supplementary files (e.g., excel). Please ensure that all data files are uploaded as 'Supporting Information' and are invariably referred to (in the manuscript, figure legends, and the Description field when uploading your files) using the following format verbatim: S1 Data, S2 Data, etc. Multiple panels of a single or even several figures can be included as multiple sheets in one excel file that is saved using exactly the following convention: S1_Data.xlsx (using an underscore).
2. Deposition in a publicly available repository. Please also provide the accession code or a reviewer link so that we may view your data before publication.

*Our data has been deposited in a publicly available repository hosted by Open Science Framework and available at:* [*https://doi.org/10.17605/OSF.IO/E58WS*](https://doi.org/10.17605/OSF.IO/E58WS)*. The two files that are needed to reproduce our figures can be found within “4_automated-evaluation/data/tidy_data/indicators_all.csv” and within “4_automated-evaluation/data/tidy_data/pmcoa-meta-data_select.csv.” Note that the code is constructed in such a way as to automatically recognize which data files to use (i.e. the user will not have to determine this).*

Regardless of the method selected, please ensure that you provide the individual numerical values that underlie the summary data displayed in the following figure panels as they are essential for readers to assess your analysis and to reproduce it: Figs 1AB, 2, 3AB, 4, 5AB, 6, S1. NOTE: the numerical data provided should include all replicates AND the way in which the plotted mean and errors were derived (it should not present only the mean/average values).

*All of our data can be found on OSF (please see our response to Comment #2 above). Upon downloading our data from OSF and code from GitHub, users should be able to reproduce our exact figures by running the file called “paper-from.code.Rmd.”*

IMPORTANT: Please also ensure that figure legends in your manuscript include information on where the underlying data can be found, and ensure your supplemental data file/s has a legend.

*We have updated our manuscript to include the exact link to our data on OSF and code on GitHub. We have also updated our GitHub to help anyone interested understand how to run our code to obtain our results.*

Please ensure that your Data Statement in the submission system accurately describes where your data can be found.

# REVIEWER COMMENTS

## Reviewer #1

[identifies himself as Adrian Barnett]

This was an interesting paper describing a worthwhile task of automating assessing research papers for key transparency criteria. Transparency is a vital tool for improving the currently low quality of much medical research. Having these statistics easily available will make it possible to identify researchers/journals/institutions that are doing relatively well or badly.

The authors have put a lot of hard work into developing the algorithms to classify papers, including robust validation by hand. It is an impressive achievement to get such high sensitivity/specificity figures. I think these tools will be useful for research and will likely be improved as people use them.

*We would like to thank the reviewer for the kind comments. We too hope that others will use and most importantly improve on our tools and make their work openly available.*

Sadly, I could not get the R package to work on some test papers that I downloaded. A vignette and/or more instructions would be useful. My faith in the tools would be greatly increased if I could see them working on my own system.

*We absolutely agree. We have now included functions that make our package more user-friendly and created a vignette that can be found on GitHub here: https://github.com/serghiou/rtransparent/tree/master/vignettes.*

The results showed surprisingly high figures for transparency, particularly on data sharing. There were also encouraging increasing trends over time for conflicts of interest. The other indicators were relatively flat, demonstrating - yet again - how science needs to improve.

The data sharing indicator includes researchers promising to make their data available, but researchers often fail to follow-through on these promises and the best level of data sharing is a link to the actual data. However, at least this is able to document the presence of a promise and there may be later consequences, such as when a researcher claims no conflict of interest but then one is found.

*Thank you for sharing this comment. We should clarify that the Data and Code sharing algorithms try to identify sharing of new data and avoid statements that do not immediately share data or code (e.g. “upon request” statements). This was previously noted in the Results section (page 11 - note that page numbers refer to the untracked manuscript, not the tracked one) as “Note that, even though in the manual assessment, Data sharing and Code sharing indicators capture any statement about data or code availability, in the automated assessment we only capture newly generated open raw data or code, to align with previous work in this field [29].” We have now updated our Methods section to provide additional clarity (Page 31): “It should be noted that, unlike in our manual assessment, these algorithms were designed to capture data or code sharing where new data is actually made available, and avoid claims of data sharing, such as “upon request.””*

Minor comments

- You could simply round percents over 10% to whole digits, except the sensitivity/specificity percents near 100. See "Too many digits: the presentation of numerical data", DOI: 10.1136/archdischild-2014-307149

*Yes, we had considered rounding percentage figures, but initially decided against it to avoid inconsistencies with figures in which a decimal place was meaningful. However, given your remark, we have now rounded the majority of percentage figures, apart from a few occasions in which we felt the decimal place was necessary. We also defer to the journal and what style they would like to pursue.*

- Some false positives could well occur for articles that are opinion pieces about data or code sharing.

*Thank you for this perceptive comment. As we also answered in a similar comment by another reviewer, we had encountered that problem in the context of the Data, Code and Registration indicators. We initially tried mitigating this issue by using the PubMed Central (PMC) XML labels for the type of article and then selecting only for research articles. However, we realized that these labels were inconsistent (e.g. research articles labelled as reviews, letters referring to both research or review articles, etc.), for which reason we abandoned that strategy. Instead, we created labelling functions that employ contextual cues, which was beneficial but exhausted what can efficiently be done using our declarative approach. Future probabilistic approaches should be able to address this issue more appropriately. Nevertheless, our algorithms were evaluated on ANY publication they may be encountered on PMC, which means that the reported performance incorporates their performance within opinion pieces. We have now explicitly incorporated this limitation within our limitation section.*

*Page 24, Discussion (Limitations): “Finally, even though we have assessed all open access articles on PMC, some of these articles cannot be expected to share Data, Code or a Protocol registration (e.g. an opinion paper). Even though future work is necessary to identify such contextual characteristics as the type of article, we have mitigated their impact by incorporating their existence within the evaluation of our algorithms and, where meaningful, providing assessments of indicators within research articles alone. However, it should be noted that many of the articles labelled by OCC as non-research may in fact contain empirical research (see “Sources of meta-data” in Materials and Methods).”*

- An agreement statistic would be more appropriate than Fisher's test for comparing raters. E.g., Gwet's agreement statistic.

*Indeed, a more common measure of interrater reliability would have been appropriate, had the reviewers assessed the same articles. However, each reviewer assessed a different random batch of articles, for which reason we could only compare relative frequencies. We have now clarified this in our “Statistical Information” section of the Materials and Methods. As indicated in the “Manual assessment of transparency” section of our Materials and Methods, before this assessment all three reviewers went through the same 40 articles and agreed on all extracted information.*

*Page 38, Materials and Methods: “we did not use common measures of inter-rater reliability because each reviewer assessed a different random batch of articles.”*

- You mention standardisation at the NLM, it would also be useful to encourage journals to use standard wording around data sharing, conflicts, etc. Although that is a much harder task.

*Thank you for this comment. Our Discussion read: “this is a good opportunity for publishers and the National Library of Medicine (NLM) to standardize reporting of such indicators.” We have now modified this sentence to also include journals to stress that we should all work towards standardization.*

*Page 22, Discussion:“most journals use very different language when referring to such indicators.”*

## Reviewer #2

[identifies herself as Quinn Grundy, University of Toronto]

The authors aimed to develop an algorithm capable of assessing multiple indicators of transparency (conflict of interest and funding disclosures) and reproducibility (data sharing, code sharing and protocol registration statements) across the biomedical literature indexed in PubMed at scale. Their algorithm largely demonstrated accuracy, sensitivity and specificity across indicators, with the exception of indicators related to data sharing and code sharing. Overtime, they demonstrate that the proportion of articles including an indicator has increased for all indicators over the study period and particularly, conflict of interest and funding disclosures. Overall, the paper was well-written and comprehensive in its reporting.

*This is an accurate representation and thank you for your kind words.*

I make the following comments, but please note that my expertise relates to conflicts of interest and research integrity and I am not qualified to evaluate the methods related to the building or testing of the algorithm.

The authors state that the chief contribution of the paper is the algorithm and corresponding data set, which they make open access. However, I think the contribution of this work and the algorithm/data set could be more explicitly stated both in the introduction and in the discussion.

*Thank you for your guidance. We have now updated our Introduction and Discussion sections to reflect these suggestions. Note that all Page numbers refer to the untracked version of our manuscript, not the tracked one.*

*Page 6, Introduction: “By doing so, we are able to, for the first time that we know of, map all of these indicators across publishers, journals, countries and disciplines. Finally, we make our tools and data openly available, hoping that these will serve as a unifying foundation upon which future work will build and expand.”*

*Page 23, Discussion: “and map such indicators across time, countries, journals, publishers and disciplines” and “understand where and how critical resources may be invested in improving transparency in the biomedical literature.”*

In the introduction, the authors make a case for achieving transparency and more open science. Transparency is often the reflexive reaction and 'fix' suggested for all issues related to research integrity, however, it really is a means to an end. The introduction could be strengthened by distinguishing and making more explicit the relationships among the key concepts (transparency, reproducibility and replication) and their relationship to research integrity.

*Thank you for this suggestion. We have now updated our Introduction to address this concern:*

*Page 5, Introduction: “Such transparent practice, among others, facilitates reproducibility by providing the data and code required to rerun reported analyses and promotes replicability by providing a detailed account of the methods and protocols employed.”*

In the discussion, the authors state that their work demonstrates that "using automated approaches to study and understand the biomedical literature is possible and can yield insights over and above those possible with manual assessments of small random samples." However, they also note that uptake of simple interventions such as XML tagging has been highly limited. The authors might consider more explicitly denoting the audience for this work (researchers?) or suggesting particular implementations (or noting that this work remains exploratory). For example, could this dataset be used by journals or fields of science to benchmark their transparency practices? In what ways could these tools be used to understand actual transparency and reproducibility rather than indicators?

*Thank you for these suggestions. We have now written a new paragraph within our discussion to explicitly denote particular implementations.*

*Page 21, Discussion: “Indeed, we hope that our tools, data and results set a precedent that can unify, inspire and inform future research and practice. Our tools have been open sourced as an R package so that other teams or individuals may apply them within their collection of articles, improve on their current implementation and expand them to indicators of transparency not currently captured; all within one unified package. Our data, as indicated above, provide a foundational yet flexible map of transparency to expedite further research in the area, such that future researchers may investigate these indicators across different definitions or different subsets of the literature, or integrate these data within their own data pipeline such that any re-extraction is avoided. Finally, our results help us monitor the status quo and suggest the existence of different practices across publishers and disciplines, providing an opportunity to reflect and learn from the experience of each other.”*

The findings of this study also need to be contextualized beyond the 'biomedical' literature. The authors included all articles indexed in PubMed; from the Tables of study characteristics, it is apparent that articles from a number of fields of study including humanities, 'health', and social sciences were included. For a number of these fields of study, empirical articles may include studies for which some of the indicators are inappropriate, namely data sharing, code sharing and protocol registration (e.g. qualitative research, data that cannot be anonymized, analysis that does not involve code, designs for which you can't register protocols nor are reproducible). The authors might comment on when/how the algorithm is most applicable, where it has limited value, and where it can be universally applied (e.g. funding disclosures). Further, how did the authors determine what was considered "empirical" research? Figure 5 in particular should be contextualized.

*Thank you for this perceptive comment. As we also answered in a similar comment by reviewers #1 and #3, we had encountered that problem in the context of the Data, Code and Registration indicators. We initially tried mitigating this issue by using the PMC XML labels for the type of article and then selecting only for research articles. However, we soon realized that these labels were very non-standard (e.g. research articles labelled as reviews, letters referring to both research or review articles, etc.), for which reason we abandoned that strategy. We then decided to create labelling functions that employ contextual cues, which was beneficial but exhausted what can efficiently be done using our declarative approach. Future probabilistic approaches should be able to address this issue more appropriately by identifying the type of article. Nevertheless, our algorithms were evaluated on ANY publication they may encounter on PMC, which means that the reported performance incorporates their performance within opinion pieces. We have now explicitly incorporated this limitation within our limitation section: “Finally, even though we have assessed all open access articles on PMC, some of these articles cannot be expected to share Data, Code or a Protocol registration (e.g. an opinion paper). Even though future work is necessary to identify such contextual characteristics as the type of article, we have mitigated their impact by incorporating their existence within the evaluation of our algorithms and, where meaningful, providing assessments of indicators within research articles alone. However, it should be noted that many of the articles labelled by OCC as non-research may in fact contain empirical research (see “Sources of meta-data” in Materials and Methods).”*

*In terms of empirical research, this label was determined and taken by the NIH Open Citation Collection (OCC), which itself bases this definition on data provided from PubMed. We have now added further detailed explanations as indicated below.*

Minor comments

1. For manual assessment, you report, "and a substantial portion mentioned use of public funds". Do you mean that this portion disclosed support from public funding sources only? (i.e. publicly-funded research?) Could you rephrase to clarify?

*Thank you for this. Yes, this referred to exclusive support from public sources and it has been rephrased:*

*Page 9, Results: “a substantial portion disclosed exclusive support from public sources”*

2. Figure 1 - how were these data combined with the data from the previous studies? Also, I did not understand the rationale for combining these data sets.

*Our data were merged with similar data from two older articles previously published by members of this team (10.1371/journal.pbio.2006930 and 10.1371/journal.pbio.1002333). Our data were combined with previous data because our data cover years 2015-2018 (in detail), whereas previous data cover years 2000-2015 and 2015-2018 (in less detail). We have now rephrased the figure caption.*

*Page 9, Fig 1: “Indicators of transparency and reproducibility across time on the basis of manual assessment. Proportions are displayed as a 4-year centered moving average. These graphs merge data from this study on 349 research articles (2015-2018) with similar data from two previous studies on another 590 PubMed articles (2000-2018) [20,22] for a combined total of 939 research articles.”*

3. It was my understanding that PubMed/NLM instituted a change that required the inclusion of conflict of interest and/or funding statements in the meta-data. Can you comment on this policy change? Does this refer to the fact that many journals are not making use of the XML tags? Could this account for the notable increase in prevalence of COI statements?

*The only such policy we are aware of is for inclusion of Conflict of Interest Disclosure in Supplements or Special issues, which came in effect in 2007 as denoted here:* [*https://www.nlm.nih.gov/bsd/policy/conflict_of_interest.html*](https://www.nlm.nih.gov/bsd/policy/conflict_of_interest.html)*. As far as we can tell, this is unrelated to XML tags and no mention of it is made by NLM in its XML tagging guidelines as shown in the following link:* [*https://www.ncbi.nlm.nih.gov/pmc/pmcdoc/tagging-guidelines/article*](https://www.ncbi.nlm.nih.gov/pmc/pmcdoc/tagging-guidelines/article/dobs.html#dob-coi)*.*

*Our work only examined mentions of indicators in the main text, not in supplements. We have now clarified this by updating the Methods and Results sections.*

*Page 24, Methods - PubMed Central Open Access Subset (PMCOA): “These articles are made available under a Creative Commons or similar license and their full text (but not their supplements) may be downloaded in bulk (as XML files) and used for research; these are the publications that were used to estimate the open access-wide biomedical literature degree of transparency.”*

*Page 13, Results - Automated assessment of transparency: “Note that these records did not include any supporting information to the main text.”*

4. In Figure S2. I did not understand what was meant by "both" correct, "both" wrong? Could you explain?

*The caption to S2 Fig has been re-written to clarify these definitions. The text of interest reads: “Both correct = this text was labelled correctly by both algorithms; Updated correct = this text was labelled correctly only by the updated algorithm; Both wrong = this text that was labelled incorrectly by both algorithms.*

5. Open Citation Collection - how do they define "research articles"?

*They use a definition based on article type categorizations by NLM, as explained here:* [*https://icite.od.nih.gov/user_guide?page_id=ug_data#article*](https://icite.od.nih.gov/user_guide?page_id=ug_data#article)*. We have now added the following clarification in the “Sources of meta-data” section of Materials and Methods.*

*Page 25, Materials and Methods - Sources of meta-data “We also used OCC to extract whether an article is considered a research article or not - this is based on definitions by PubMed and considers articles such as those labelled by PubMed as journal articles, randomized controlled trials or observational studies as research articles, and articles such as those labelled as editorials, news or comments as non-research [38] - it should be noted that in our work we identified that PubMed annotations do not always agree with PMC and that 47,631 articles labelled by PMC as “research articles”, were classified as a non-research type by PubMed (and thus, iCite) - in these cases, we maintained the definition of research by iCite to remain internally consistent.”*

6. The authors state, "Note that unlike in our manual assessment, the numbers reported in this section refer to the entire literature on PMCOA, not merely research articles; all analyses were repeated in research articles alone with no meaningful changes." It would seem that for the vast majority of records (e.g. editorials, reviews, commentaries, letters etc.) that Data sharing and Code sharing would be irrelevant. Could the authors comment on this finding?

*There are two primary reasons this may be the case: (a) the majority of articles (~85%) are labelled by OCC as research articles, which means that removing non-research articles only has a minor impact and (b) many articles (potentially up to 20%) labelled by OCC as non-research, in fact include empirical research. This is because, even though OCC uses data available on PubMed to adjudicate research vs non-research (as indicated above), it appears that many articles receive a different label by PMC (PubMed Central), such that for example an article may be labelled as a “Review” on PubMed, but as a “Research article” on PMC.*

*We have now clarified this by including this sentence in our first use of the research articles concept (Page 13, Results): “Note that open access records from PMC did not include any supporting information and that the classification of OCC into research articles is imperfect (see Sources of meta-data in Materials and Methods).”*

*We have also added this sentence at the end of the Limitations paragraph (Page 23): “However, it should be noted that many of the articles labelled by OCC as non-research may in fact contain empirical research (see “Sources of meta-data” in Materials and Methods).”*

*And we have added this in the Materials and Methods (Page 25): “it should be noted that in our work we identified that PubMed annotations do not always agree with PMC and that 47,631 articles labelled by PMC as “research articles”, were classified as a non-research type by PubMed (and thus, iCite) - in these cases, we maintained the definition of research by iCite to remain internally consistent.”*

7. With funding, could discrepancies be because work was actually unfunded (and thus no disclosure)? With funding, absence of disclosure may indicate no funding. Or, conflation of COI and funding.

*Indeed it may indicate no funding, for which reason we refer to the presence of Funding as the presence of “Funding statements”, because we can only tell whether a statement exists, not if this statement is necessarily complete or truthful. This is already part of our limitations (page 22): “Third, algorithms in general do not guarantee that the statements identified are either accurate, full, or true. This is why we have designed our algorithms to not only extract text, but also indicate why an article was labelled as including information on an indicator. We hope that future work will study these questions further.” In terms of conflation with COI, we agree that this is an important issue and that different researchers understand different statements differently - we have addressed this by explicitly indicating which labelling functions were responsible for a COI/Funding label such that researchers interested in using our dataset can flexibly create their own definitions. This is also part of our Discussion (Page 21): “As such, they can facilitate other teams in building systems on top of this with more nuanced definitions of COI without having to retrain and re-calibrate complex black box systems.”*

8. When you state, "Similarly, no publisher reports Data sharing in more than ~75% of publications. Is this among research articles? Or all publications (i.e. journals)?

*Publications were used to refer to articles that were labelled by PubMed/OCC as research or non-research. We have now replaced all mentions of publications with “articles” and clarified at the beginning of the paragraph that articles refer to “research or non-research.” We have also further clarified the wording in the paragraph. At the end of the paragraph we denote that similar analyses in articles labelled by OCC as research did not lead to meaningful changes.*

## Reviewer #3

This manuscript does an excellent job of successfully demonstrating an automated approach for measuring and characterizing the transparency of the biomedical literature. In addition to identifying important trends by time, discipline, and indicator type, the authors present a set of open algorithms and data that are intentionally designed/presented to facilitate future and more nuanced research questions.

Only a few minor revisions are recommended:

1. The authors describe alternative automated approaches for assessing transparency, but do not reference SciScore. While SciScore focuses on indicators of rigor, I believe it's shared attention to the problem of reproducibility is potentially noteworthy.

*Thank you very much for flagging this very interesting work. Even though we recently came across SciScore, this did not exist when we first wrote our manuscript. We have now updated our Introduction.*

*Page 6, Introduction: “Another recently published tool called SciScore, uses a machine learning method known as conditional random fields to identify measures of rigor (e.g. randomization, blinding, power analysis) across the open access literature on PubMed and create a score of rigor and transparency. However, this tool does not include indicators of transparency (e.g. data or code sharing), did not openly share article-specific data and the underlying code was not made available (Menke et al. 2020).”*

2. While the authors identify the five indicators of transparency their work addresses, they do not offer a rationale for why these indicators were chosen. It would be helpful for readers who are less familiar with this field to provide a brief, but explicit explanation.

*Thank you for this comment. This work builds upon our previous evaluation of transparency and reproducibility indicators (10.1371/journal.pbio.2006930 and 10.1371/journal.pbio.1002333), which were developed based on the recommendations from a team of experts. We have now added the updated Introduction.*

*Page XX, Line “In 2014, a team of experts outlined a number of recommended indicators of transparency that may be monitored by interested stakeholders to assess and promote improvements in biomedical research [21].”*

3. Results section, P1, S1 - consider replacing alternative with additional.

*We have now replaced “alternative” with “all other.”*

4. In the manual review of PubMed articles, "data upon request" like statements were, I believe, considered positively indicative of transparency; however, the automated approach applies a stricter criterion. Consider briefly describing the reason for this change.

*To align with previous work in this field (Riedel et al 2020), our automated assessment only captured newly generated open raw data or code. We have now updated our Results section to reflect this justification:*

*Page 10, Results: “Note that, even though in the manual assessment, Data sharing and Code sharing indicators capture any statement about data or code availability, in the automated assessment we only capture newly generated open raw data or code, to align with previous work in this field (Riedel et al. 2020).”*

5. It would be helpful to reference the criterion OCC uses to identify "research" articles: <https://icite.od.nih.gov/user_guide?page_id=ug_data#article>.

*This is a great point and other reviewers have also made it. We also really appreciate you being so kind as to share this link with us. We have now cited the link in our methods.*

*Page 25, Methods“We also used OCC to extract whether an article is considered a research article or not - this is based on definitions by PubMed and considers articles such as those labelled by PubMed as journal articles, randomized controlled trials or observational studies as research articles, and articles such as those labelled as editorials, news or comments as non-research [38] - it should be noted that in our work we identified that PubMed annotations do not always agree with PMC and that 47,631 articles labelled by PMC as “research articles”, were classified as a non-research type by PubMed (and thus, iCite) - in these cases, we maintained the definition of research by iCite to remain internally consistent.”*

6. The authors' description of their automated method for identifying pre-registered protocols strikes me as focused on repositories and naming conventions specific to clinical trials and human-subjects research; however, a significant portion of the analyzed papers are pre-clinical. If my interpretation of the methodology is accurate, consider addressing the focus and limitations of this approach. If my interpretation is inaccurate, revise the methods to provide non-clinical examples.

*Our Protocol registration algorithm was designed to specifically identify all registries that we know of (i.e. those listed by the International Trial Registry of the WHO and PROSPERO), as well as identify mentions of registration to registries that we did not know of. We have now clarified this by adding the phrase “regardless of registry” in our description of this algorithm.*

## Reviewer #4

[identifies himself as Fabian Held]

Thank you for giving me the opportunity to review this manuscript titled "Assessment of transparency indicators across the biomedical literature: how open is open?". It presents an open-source, automated approach to identify five indicators of transparency in biomedical research literature, including conflicts of interest disclosures, funding disclosures, data sharing, code sharing and protocol registration. The manuscript describes the development and evaluation of algorithms that automatically identify relevant parts of a publication. Furthermore, it demonstrates that this can be achieved at the scale of millions of articles by analysing the entire open access biomedical literature of 2.75 million articles on PubMed Central.

The insights and tools presented here constitute a substantial advancement in our ability to monitor transparency in research and encourage reproducible research practices and the assessment of risk of bias.

*This is an accurate description of our work and thank you for your comments.*

- The work presented is complex: five different algorithms were developed, evaluated, and employed to report findings across millions of articles. Unfortunately, development for CoI and funding extraction deviated from the train-validate-test paradigm. The manuscript uses several subsets of data with various qualifications across multiple stages and the current explanation of their evaluation process is hard to follow. In fact I am unclear how the final algorithms were evaluated, how their test sets were coded and how (or if) the test sets are related across algorithms. One example is the following statement from p.31: "To do so, we set aside 225 articles and then redeveloped the algorithms in the remaining data. […] COI disclosures were evaluated in 100/4792 predicted positive and 226/1225 predicted negative (one extra was mistakenly included and not subsequently removed to avoid bias). Funding disclosures were evaluated in 100/5022 predicted positive and 225/995 predicted negative." The evaluation process in the methods section should be rewritten for clarity. Possibly a schematic diagram of the process coding and data (sub)sets could be helpful.

*We resonate with the reviewer’s thoughts about our description of validation. We have now reworded our description of validation and reorganized the paragraphs into titled sections (see pages 32-34 of the untracked manuscript). We have also included a flowchart as a supplement to illustrate our basic approach (S3 Fig).*

- Furthermore it would be instructive for readers to mention earlier in the paper what kinds of algorithms are discussed here. These are not machine learning or statistical algorithms, but largely rules-based, using regular expressions.

*We have now modified our last paragraph of the Introduction (Page 6) to say “rule-based tools” and in the Results we modified the first mention of our algorithms to say “by using a rule-based approach of regular expressions.”*

- In the results sections I would recommend adding a column for totals to Fig5a and I am unconvinced that Fig5b is an effective visualisation. Sizes of very small circles are difficult to interpret and the added colour coding for fields of science distorts the visual effect of size. Maybe shading could be used for estimated prevalence and a coloured overlay could indicate field?

*We have now added a Totals column to Fig 5a. We also appreciate and thank you for your feedback about Fig 5b. However, this format has already been used to publish these maps elsewhere (see the Scientific Data article from 2020 here:* [*https://www.nature.com/articles/s41597-020-00749-y*](https://www.nature.com/articles/s41597-020-00749-y)*) and we believe that maintaining consistency is important. Additionally, in this publication we only aim to show general trends rather than delve into specific circles - should we pursue this approach, we believe that implementing your feedback would be valuable.*

- Finally, the labelling in the third table in S4 Text. Performance metrics speaks of "manual assessment" which strikes me as misleading as this is a table of expected values.

*Thank you for your very perceptive comment. We have now modified the final table to say “Manual/Automated assessment (expected counts)” and also included the following sentence: “Note that these numbers represent statistics of expected counts, not true counts.”*
