# Supplementary material for: Assessment of transparency indicators across the biomedical literature: How open is open?
Source: PLoS Biol. 2021 Mar 1;19(3):e3001107. doi: 10.1371/journal.pbio.3001107 (PMC7951980; doi:10.1371/journal.pbio.3001107)
Supplement: Attachment — Submitted filename: response-to-reviewers.docx [file pbio.3001107.s019.docx]

Response to reviewers

# Editor

a. Please could you address the remaining points raised by reviewer #1? Some pertain to the Github deposition, and some to the manuscript itself.

*We thank the peer reviewer for his meticulous work and for diligently working through our R package. We have now responded to all of his feedback.*

b. Many thanks for making the data underlying the Figures available in your OSF deposition. However, please could you cite the URL in all relevant main and supplementary Figure legends, e.g. "The data underlying this Figure may be found at http://www.doi.org/10.17605/OSF.IO/E58WS".

*The following phrase has now been added to the Figure legends of all relevant figures in the manuscript and supplement: “The data underlying this Figure can be found on OSF at http://www.doi.org/10.17605/OSF.IO/E58WS.”*

# Reviewer 1

This is an important and worthwhile piece of research and the authors have answered my previous comments.

The authors have added a vignette and I was able to run their code on some papers and see it working. I have some minor quibbles with the vignette, but these are not a bar to publishing the paper in its current form.

There was no rendered version of the vignette online, so whilst I could run the vignette I could not check my results against the authors.

*We thank the author for alerting us to this. As per best practice, R packages do not maintain a knitted copy of the vignette on their GitHub repository. Instead, vignettes are built and available in the GitHub repository of the CRAN. However, our package has not yet been made available on CRAN, which is why there is no available knitted vignette. Users can see the knitted vignette by installing our package using `devtools::install_github(build_vignettes = T)` and then `vignette(“rtransparent”)`. We have now modified the README.md page of our package to direct users to the rendered version of the vignette.*

I was able to get the PDF version working, but not the version using XML. E.g., for the example used in the vignette I got the following when using the PDF route:

$ article	<chr> "10.17605/OSF.IO/E58WS"

$ is_open_data	<lgl> TRUE

$ open_data_category <chr> "field-specific repository, general-purpose repository, data availability statement"

$ is_open_code	<lgl> FALSE

$ open_data_statements <chr> "\"all data are made available on a public repository (openfmri accession number ds000202).; \"raw data derived from this anal...

$ open_code_statements <chr> ""

And the following when using the XML route:

$ pmid	<chr> ""

$ pmcid_pmc	<chr> ""

$ pmcid_uid	<chr> ""

$ doi	<chr> ""

$ filename	<chr> "PMID30457984.xml"

$ is_research <lgl> FALSE

$ is_review	<lgl> FALSE

$ is_success <lgl> TRUE

*Thank you very much for trying to use our package and alerting us to this! Unfortunately, PMC uses a slightly different structure for XML documents retrieved from different services. As such, using the same functions we used to process PMC XML documents obtained from the PMCOA batch download (as presented in our manuscript) for an XML document downloaded from the FTP service (presented in the vignette), did not work. We have now updated the code so that it is robust to such changes, by standardizing the root node of the XML file.*

**Minor comments**

- Table 2, not really a "distribution" as it's simply the number and percent. A couple of large numbers are missing commas, e.g., Medicine row.

*Thank you for this comment and we agree. We have now renamed our table into: “Meta-data across all 2,751,420 open access publications on PubMed Central (PMCOA)” and we have reformatted all numbers to include commas.*

- "Funding disclosure information in 67.9% (67.6-68.3%)," missing "95% CI"

*Thank you for this. We have now added the missing “95% CI”.*

- It's hard to see small countries in Figure 4. A table of the top 10 countries might be more useful.

*We appreciate this comment and we had indeed considered offering such a table ourselves. However, we decided against that course of action because we wanted to avoid creating yet another, potentially quite misleading, “ranking” of countries. This is why we also did not refer to any specific countries in our figure legend. Instead, these maps primarily aim to illustrate the distribution of indicators of transparency across the world and suggest that, overall, the prevalence of these indicators is homogenous across countries.*

- It was useful to have examples of where the code worked and did not work (Fig s2).

*Thank you very much for appreciating our figure! We believe so as well. This tool is far from perfect and we wanted our readers to appreciate that and to hopefully feel compelled to work on improving this open-source tool.*

- The help files for rtransparent need some more detail to tell users what the variables are. For example, the command to search for funding returns 52 variables, but the help file does not detail what these are.

*We apologize for this inconvenience. We have now added further descriptions to each function to clarify the meaning of each variable within the dataframe returned by each function. For example, we have now updated the description for `rt_all_pmc` to: “A dataframe of results. It returns the unique identifiers of the article, whether each of 3 indicators of transparency (COI, Funding or Registration) was identified, the relevant text identified, whether it was identified through a dedicated XML tag (such variables include "pmc" in their name, e.g. “fund_pmc_source”) and whether each labelling function identified relevant text or not. The labelling functions are returned to add flexibility in how this package is used; for example, future definitions of Registration may differ from the one we used. If a labelling function returns NA it means that it was not run.”*
